# Blood eosinophils as predictors of asthma exacerbations in adults: converging evidence from mendelian randomization, single-cell transcriptomics, and a two-center longitudinal cohort

**DOI:** 10.3389/fimmu.2026.1927894

**Published:** 2026-09-22

**Authors:** Delong Huang, Zhengrong Bao, Haoshu Zhong, Junhao Wu, Peng Zhang, Qiang Zhang, Shaojie Luo, Chunfeng Liu, Qiang Ren

**Affiliations:** 1 School of Clinical Medicine, Southwest Medical University, Luzhou, China; 2 Department of Neurosurgery, The Second Affiliated Hospital of Soochow University, Suzhou, China; 3 Department of Neonatology, Children’s Medical Center, The Affiliated Hospital, Southwest Medical University, Luzhou, China; 4 Department of Perinatology, The Affiliated Hospital, Southwest Medical University, Luzhou, China; 5 Sichuan Clinical Research Center for Birth Defects, Luzhou, China; 6 Department of Hematology, Huashan Hospital, Fudan University, Shanghai, China; 7 Department of Nuclear Medicine, Sichuan Provincial People’s Hospital, University of Electronic Science and Technology of China, Chengdu, China; 8 Neijiang Health Vocational College, Neijiang, Sichuan, China; 9 Department of Scientific Research, The Shapingba Hospital, Chongqing University (People’s Hospital of Shapingba District, Chongqing), Chongqing, China; 10 Department of Respiratory and Critical Care Medicine, The Affiliated Hospital, Southwest Medical University, Luzhou, China

**Keywords:** asthma, eosinophil, machine learning prediction model, Mendelian randomization, single-cell transcriptomics

## Abstract

**Background:**

Asthma is a common chronic respiratory disease that imposes a heavy burden of morbidity and mortality in most countries. Eosinophils have become major therapeutic targets for severe eosinophilic asthma and other eosinophil-associated diseases. This study investigated the role of eosinophils in asthma through three complementary approaches: (i) Mendelian randomization to test causal associations, (ii) single-cell transcriptomic analysis to explore potential biological mechanisms underlying elevated blood eosinophil counts, and (iii) clinical data analysis and machine learning modelling to evaluate their predictive value in clinical practice. These analyses demonstrated the potential value of eosinophils in clinical applications.

**Methods:**

We conducted three complementary but methodologically independent analyses in adult asthma patients. First, a two-sample Mendelian randomization analysis using publicly available GWAS summary statistics was performed to assess whether genetically predicted eosinophil count and eosinophil percentage are causally associated with asthma susceptibility. Second, a publicly available peripheral blood mononuclear cell scRNA-seq dataset (GSE248688), which contrasts asthma patients with healthy controls, was used to characterize transcriptomic differences and intercellular communication in the potential biological mechanisms for increased blood eosinophil counts in adult asthma. Third, using retrospectively collected data from two adult asthma cohorts (SWMU, n = 370, training/internal validation; Chongqing, n = 180, external validation), we developed and externally validated machine learning models for 12-month exacerbation risk. All predictors were measured at a clinically stable baseline visit preceding the 12-month outcome window.

**Results:**

The Mendelian randomization analysis provided genetic evidence that eosinophil count is causally associated with asthma susceptibility (IVW: OR 1.005, 95% CI 1.002–1.008, P < 0.001), whereas the signal for eosinophil percentage was small and borderline (IVW: OR 0.984, 95% CI 0.970–0.998, P = 0.027) and directionally inconsistent with the clinical data, most plausibly reflecting the compositional nature of a percentage measurement. Overall, eotaxins and type 2 inflammation provide a plausible explanation for increased blood eosinophil counts in asthma patients. Further analysis revealed that the platelet subcluster in the asthma group highly expressed CCL26 (eotaxin-3). Upregulated transcriptional activity of GATA3 and MAF was observed in CD14^+^ monocytes. Cell-cell communication analysis indicated network remodeling. Specifically, three cell types—CD14^+^ monocytes, platelets, and hematopoietic stem and progenitor cells (HSPCs)—simultaneously showed upregulated sender activity for MIF signaling. The five machine learning models achieved external-cohort AUCs ranging from 0.718 to 0.769 (RF 0.763, XGBoost 0.769, LR 0.755, SVM-RBF 0.905, KNN 0.733), with substantially overlapping 95% CIs; a parsimonious three-variable benchmark model achieved a comparable AUC of 0.892.

**Conclusion:**

Peripheral blood eosinophils may play an important role in asthma. They are especially promising as potential clinical predictors of asthma exacerbation. However, the mechanisms underlying their increase are complex. We speculate that three pathways may be involved: bone marrow release, peripheral survival, and peripheral activation. Therefore, both the clinical utility and the specific biological mechanisms of peripheral blood eosinophils still require further validation.

## Introduction

Asthma is a common chronic respiratory disease, with approximately 260 million people affected worldwide. It is one of the most common chronic diseases in both high-income and low- and middle-income countries. Asthma now imposes a significant burden of morbidity and mortality in most countries, along with a high economic burden (1). While asthma exhibits familial clustering, its increased prevalence since the 20th century is more likely attributable to changes in the environment and lifestyle (2). Asthma is characterized by common symptoms (i.e., wheezing, coughing, and shortness of breath) as well as airway obstruction, which is typically reversible. Furthermore, according to the Global Initiative for Asthma (GINA) general definition, asthma is a heterogeneous syndrome typically characterized by chronic airway inflammation (3). Although respiratory symptoms appear similar, large-scale stratified studies indicate that asthma encompasses multiple phenotypes, which may be driven by different underlying pathophysiological mechanisms (i.e., subtypes) (4, 5). For example, asthma exhibits significant differences in age of onset (childhood-onset vs. adult-onset), triggers (infections or allergies), type of airway inflammation (eosinophilic, neutrophilic, paucigranulocytic, or mixed), and clinical course (acute exacerbation, progressive, or stable) (2, 6). Consequently, an increasing number of studies have suggested that asthma requires more specific classification criteria (7, 8) to further improve its assessment and treatment (7, 9). Among these, eosinophils play a crucial role in the diagnosis and treatment of asthma subtypes (10).

A common phenotype of asthma, known as eosinophilic asthma, is characterized by the presence of eosinophilia (11, 12). Type 2 helper T (Th2) lymphocytes or their signature cytokines play a crucial role in driving Type 2 (T2) asthma symptoms. Th2 cells are specialized producers of key cytokines involved in allergic and type 2 inflammation, including interleukin (IL)-4, IL-13, and IL-5. These cytokines promote bronchial hyperresponsiveness, IgE and mucus production, and eosinophilia (13, 14). Since blood and induced sputum are relatively easy to obtain, the stratification of patients into T2 and non-T2 phenotypes is typically based on the presence or absence of elevated blood or airway eosinophil counts (15, 16). Eosinophils play a crucial role in asthma pathogenesis, and growing evidence further suggests that blood eosinophils are important in asthma exacerbations (17, 18). Activated eosinophils lead to major pathological changes during both the stable and exacerbation phases of asthma (19). For example, CD62L expression on eosinophils is positively correlated with the clinical severity of severe eosinophilic asthma; *in vitro*, CD62L expression on eosinophils is regulated by IL-5 and anti-IL-5 (20). Furthermore, asthma is associated with excessive production of leukotrienes (LTs), including LTB4 (21). Patients with severe asthma may be highly sensitive to 5-lipoxygenase (5-LO) inhibition, which blocks the production of cysteinyl LTs and LTB4. LTB4 concentrations are elevated in the bronchoalveolar lavage (BAL) fluid of patients with severe asthma (including those with isolated eosinophilic inflammation), and these eosinophils express leukotriene A4 hydrolase (LTA4H) as demonstrated by immunohistochemistry (IHC). LTA4H expression in blood eosinophils has been confirmed by flow cytometry, IHC, and quantitative polymerase chain reaction (qPCR). Eosinophils express LTA4H transcripts and proteins and can be stimulated to secrete LTB4 (22).

Therefore, eosinophils may be one of the potential biomarkers of asthma exacerbations. Studies have found that patients with blood eosinophil counts ≥300 cells/μL and fractional exhaled nitric oxide (FeNO) ≥25 ppb have a higher rate of acute exacerbations. Elevated blood eosinophil counts and increased FeNO levels may be associated with an increased rate of exacerbations (23). Meanwhile, Peters et al. also found that although elevated blood eosinophil counts were not observed in patients with acute asthma exacerbations, a 100-cell/μL increase in baseline eosinophil count was associated with a 9% increase in the incidence of exacerbations, and eosinophil counts could predict disease exacerbations across the entire sample (24). A meta-analysis by Mallah et al. also found that elevated eosinophil counts were associated with a higher risk of acute asthma exacerbations [odds ratio (OR): 1.31, 95% confidence interval (CI): 1.16, 1.49], particularly with asthma-related outpatient visits [OR: 1.46, 95% CI: 1.25, 1.70] and emergency department visits [OR: 1.63, 95% CI: 1.29, 2.07]. A significant association was observed starting at an eosinophil cutoff of 200 cells/μL (25). However, most studies merely noted the association between eosinophils and asthma or asthma exacerbations, without delving into the specific predictive performance of eosinophil-related indicators in asthma.

In this study, we used three approaches to investigate the role and mechanisms of peripheral blood eosinophils in asthma. These approaches were arranged in a progressive manner. They moved from causal identification, to mechanistic speculation, and finally to clinical application. First, we performed a two-sample Mendelian randomization analysis. We used publicly available genome-wide association study (GWAS) summary statistics. This analysis assessed whether genetically predicted eosinophil counts are causally associated with asthma susceptibility. Importantly, this step aimed to establish the biological feasibility of eosinophil traits as candidate markers. It was not designed to directly evaluate exacerbation risk or to validate downstream predictive models. Second, we analyzed single-cell RNA-seq data of peripheral blood mononuclear cells (PBMCs) from asthma patients. This dataset compared asthma patients with healthy controls. We inferred the biological mechanisms and signaling pathways that may drive increased eosinophil counts in the peripheral blood. Third, we turned to clinical data. We performed statistical analyses to examine the association between blood eosinophil levels and asthma exacerbation outcomes. To further demonstrate clinical utility, we constructed machine learning-based predictive models using eosinophil-related features. Overall, our study presents the role and mechanisms of blood eosinophils in asthma. The evidence spans from causal inference, through biological mechanisms, to clinical application.

## Method

### Patients and study design

This study is a multicenter retrospective cohort study in which both baseline predictors and 12-month exacerbation outcomes were extracted from existing clinical records, with the outcome window temporally following the baseline visit for each patient to preserve predictor–outcome temporality required for prognostic modelling. Asthma diagnoses for all patients were confirmed according to the Global Initiative for Asthma (GINA) guidelines (26). To ensure a clear temporal relationship between predictor variables and the outcome, all clinical, laboratory, and pulmonary function measurements — including peripheral blood eosinophil count, eosinophil percentage, FeNO, forced expiratory volume in 1 second percent predicted (FEV_1_%pred), forced vital capacity percent predicted (FVC%pred), peak expiratory flow (PEF) variability, total IgE, ACT, ACQ-5, and EQ-5D — were collected at the baseline visit when patients were in a clinically stable state (defined as no systemic corticosteroid use, no emergency visit, and no hospitalization for asthma within the preceding 4 weeks). The outcome (asthma exacerbation) was ascertained over a 12-month observation window following the baseline visit, from electronic medical records and prescription records. An asthma exacerbation was defined, in accordance with the American Thoracic Society/European Respiratory Society (ATS/ERS) statement (27), as a worsening of symptoms requiring systemic corticosteroids for ≥3 days, an unscheduled emergency department visit, or hospitalization due to asthma. Patients whose baseline visit occurred during an ongoing exacerbation were excluded to avoid reverse causation.

Inclusion criteria: (1) age ≥ 18 years; (2) diagnosis of bronchial asthma confirmed by clinical symptoms combined with pulmonary function tests and/or bronchial provocation/bronchodilator tests; (3) clinically stable at the baseline visit; (4) complete clinical and laboratory data at baseline; (5) complete follow-up data for at least 12 months. Exclusion criteria: patients with comorbid chronic respiratory diseases (e.g., chronic obstructive pulmonary disease (COPD), bronchiectasis, interstitial lung disease, or tuberculosis), active infections, malignancies, or autoimmune diseases that may affect eosinophil levels; pregnant or lactating women; patients with significant missing key data; and patients experiencing an acute exacerbation at the time of baseline measurement. This study was approved by the Ethics Committee of the Affiliated Hospital of Southwest Medical University (Ethics Approval No.: 20251104-027). This study was conducted in strict accordance with the Declaration of Helsinki.

### Mendelian randomization analysis

To investigate whether eosinophil-related traits are causally associated with asthma susceptibility (i.e., the diagnosis of asthma rather than exacerbation, as no adequately powered GWAS summary statistics for asthma exacerbation phenotypes are currently available in the public GWAS Catalog), this study employed a two-sample Mendelian randomization analysis. We emphasise that the Mendelian randomization (MR) component is intended to establish biological plausibility for the two eosinophil traits as candidate markers, and does not directly validate the downstream exacerbation-prediction model. This study defined two exposure factors: eosinophil count and eosinophil percentage, with asthma as the outcome. The GWAS summary statistic for eosinophil count (GCST90281230) and the GWAS summary statistic for eosinophil percentage (GCST90474561) were both obtained from the GWAS Catalog database (https://www.ebi.ac.uk/gwas/); The GWAS summary statistic for asthma (GCST90080134) was also obtained from this database. The GWAS summary statistics included 30,328 (28), 441,305 (29), and 387,930 (30) individuals of European ancestry for eosinophil count, eosinophil percentage, and asthma, respectively. All single nucleotide polymorphisms (SNPs) included in the analysis were independent loci (linkage disequilibrium pruning criteria: genetic distance window = 10,000 kb, r² < 0.001) and were strong instrumental variables (F-statistic > 10) (31). The F-statistic for each SNP was calculated as the square of its effect size (β) divided by the variance of the SNP-exposure association estimate (32). When the original SNP was unavailable in the outcome dataset, a proxy variant (r² > 0.8) was obtained via LDLink (https://ldlink.nih.gov/) for substitution. During data harmonization, the effect allele directions for exposure and outcome data were standardized, and palindromic SNPs and unalignable loci were removed to ensure consistency in the direction of causal effect estimates.

This study employed the inverse variance weighted (IVW) method as the primary analytical approach, combining the SNP-specific Wald ratio estimates to derive the overall causal effect. To enhance the robustness of the results, this study also employed MR-Egger regression, the weighted median method, the weighted mode method, and the simple mode method as supplementary analytical approaches. Heterogeneity was assessed using Cochran’s Q test based on the IVW and MR-Egger estimates. To detect the impact of horizontal pleiotropy on the results, the intercept term of the MR-Egger regression was evaluated; an intercept close to zero and non-significant indicates the absence of horizontal pleiotropy. Additionally, the MR-Pleiotropy Residual Sum and Outlier (MR-PRESSO) method was applied to identify and correct outliers in the IVW linear regression, thereby reducing heterogeneity in causal effect estimates by excluding SNPs that contribute disproportionately to heterogeneity. To rule out the significant influence of individual SNPs, a sensitivity analysis using the “leave-one-out” method was conducted to compare the causal effects of the remaining SNPs with the results of the full SNP analysis. Funnel plots were used to visually assess the symmetry of the distribution of causal association points for each SNP acting as an instrumental variable; a symmetrical distribution indicates minimal variation among instrumental variables. The consistency of estimation results across these multiple methods serves as supporting evidence for the reliability of causal inference.

### Single-cell transcriptomics analysis

The scRNA-seq data used in this study were obtained from the Gene Expression Omnibus (GSE248688), comprising four pooled PBMC samples (two asthma: 12 patients and two healthy control samples: 8 patients) (33). Individual patient-level demultiplexing and detailed clinical metadata (age, sex, severity, inhaled corticosteroid (ICS)/long-acting β2-agonist (LABA)/biologic use, blood eosinophil counts) were not released with the original dataset. Raw expression matrices were processed with the Seurat package (v5.0). Quality control excluded cells with fewer than 200 or more than 5,000 detected genes, UMI counts exceeding 20,000, or mitochondrial gene proportion exceeding 20%; genes detected in fewer than three cells were also removed. Data were log-normalized (NormalizeData), the top 2,000 highly variable genes were selected (FindVariableFeatures), and expression was scaled (ScaleData). Batch effects across the four samples were corrected using Harmony. Principal component analysis was performed, with the optimal number of principal components (PCs) determined by elbow plot; unsupervised clustering used shared-nearest-neighbor graphs (FindNeighbors, FindClusters at resolution 0.4), and nonlinear visualization was achieved by uniform manifold approximation and projection (UMAP). Cell type annotations were assigned based on canonical lineage markers.

DoubletFinder was applied independently to each sample (paramSweep-optimized pK, homotypic-adjusted expected doublet rate of 0.8% per 1,000 cells, capped at 10%), and doublet-called cells were removed together with a manually identified B–monocyte doublet cluster. Second, we computed module scores (AddModuleScore) for three signatures: an eosinophil signature (*CLC, PRG2, EPX, RNASE2/3, SIGLEC8, IL5RA, CCR3, MBP*) (34), a platelet signature (*PF4, PPBP, ITGA2B, GP1BA, TUBB1, GP9*) (35), and a cytotoxic-lymphocyte signature (*GZMB, GNLY, NKG7, PRF1, KLRD1, GZMA*) (36). Because most mature eosinophils get lost during the Ficoll separation of PBMCs, the follow-up analysis mainly focused on the upstream immune drivers that might support peripheral eosinophil biology at a system level. Because GSE248688 provides only four pooled samples without patient-level demultiplexing, a conventional donor-level pseudobulk analysis is not statistically feasible. We adopted a pseudo-replicate stratification strategy: within each GEO Series (GSE), cells were randomly partitioned into four pseudo-replicates (seed = 42), yielding 8 pseudo-donors per group. Within each cell type, raw UMI counts were aggregated per pseudo-replicate and analyzed by DESeq2 with log2FC shrinkage (apeglm). To remove artifactual signals, DEGs were filtered against a three-tier blacklist covering (i) structural transcripts (IG/TCR segments, histones, hemoglobins, mitochondrial genes, ribosomal proteins, unannotated lncRNAs, pseudogenes, XIST, Y-linked genes, MALAT1/NEAT1); (ii) platelet-derived ambient RNA contaminants (applied to all non-platelet cell types); and (iii) myeloid ambient RNA contaminants (applied to T, natural killer (NK) cells, and B cell analyses). DEGs were defined as |log2FC| > 1 and Benjamini-Hochberg (BH)-adjusted p < 0.05. Cell-level Wilcoxon testing (FindMarkers) was performed in parallel for orthogonal support. Systemic T2 activation was characterized by computing log2 fold changes (asthma vs control) for T2 receptors (IL4R, IL13RA1, IL5RA), signaling components (STAT6, JAK1/2/3, GATA3, MAF), chemokine receptors (CCR3, CCR4), effector genes (AREG, FCER1A), and an eosinophil-driving axis (IL5, CSF2, IL4, IL13, CCL26, CCL5, GATA3, MAF) (37). Intercellular communication was inferred using CellChat with the human CellChatDB, and pathway-level information flow was compared between groups, with the potential signaling pathways in eosinophils examined in detail via communication heatmaps and network-role plots.

### Statistical analysis of clinical data

This study is a multicenter retrospective observational cohort study with a 12-month outcome-ascertainment window following each patient’s baseline visit, enrolling 550 adult asthma patients from the People’s Hospital of Shapingba District, Chongqing (n = 180) and the Affiliated Hospital of Southwest Medical University (SWMU; n = 370). Predictor variables included age, height, weight, body mass index (BMI), absolute peripheral blood eosinophil count (×10^9^/L), eosinophil percentage, exhaled nitric oxide (FeNO, ppb), FEV_1_%pred, FVC%pred, PEF variability, total IgE (IU/mL), specific IgE class, ICS response time (Time it takes for symptoms to improve with ICS treatment/days), Asthma Control Test (ACT), Asthma Control Questionnaire-5 (ACQ-5, treated as a continuous variable from 0–6 in accordance with its original scoring convention and a minimal clinically important difference of ~0.5), ICS daily dose (µg/day, treated as continuous), EuroQol 5-Dimension (EQ-5D) index, sex, residence, wheezing symptoms, allergic rhinitis, and bronchodilator test results. Because most continuous variables failed the Shapiro-Wilk normality test, they are reported as median [Q1, Q3] and compared using the Wilcoxon rank-sum test; categorical variables are reported as n (%) and compared using Pearson χ² or Fisher’s exact test as appropriate.

The index date for each patient is the date of the clinically stable baseline outpatient visit at which all predictor variables (peripheral blood eosinophil count/percentage, FeNO, spirometry [FEV_1_%pred, FVC%pred, PEF variability], total and specific IgE, ACT, ACQ-5, EQ-5D, ICS dose, ICS response time, wheezing, allergic rhinitis, bronchodilator test) were measured. All predictors were measured at this stable baseline visit, prior to the occurrence of any exacerbation event during the subsequent 12-month window, ensuring temporal precedence of predictors over outcome as required for a valid prognostic model, notwithstanding the retrospective nature of data collection. Patients presenting with an active exacerbation or documented respiratory infection at the index visit were excluded. The retrospective follow-up window was 12 months from the index date, during which exacerbation events were ascertained from electronic medical records and prescription records. The binary primary outcome was defined as the occurrence of at least one asthma exacerbation during the 12-month follow-up window, using the ATS/ERS Task Force definition (worsening of asthma requiring systemic corticosteroids for ≥3 days, an emergency-department visit, or hospitalization) (27). Follow-up completeness was ≥95% in both cohorts; patients lost to follow-up before 12 months were excluded from the primary analysis. Pre-specified potential confounders recorded at the index visit included prior exacerbation frequency in the preceding 12 months, baseline ICS dose category, smoking status, documented biologic therapy, and documented respiratory infection at index (the latter used as an exclusion criterion). Because retrospective extraction of biologic-therapy status and detailed prior-exacerbation history was incomplete in a substantial proportion of the two-center cohort—reflecting the pragmatic nature of routine outpatient records—we did not perform a formal confounder-adjusted sensitivity analysis restricted to fully documented subgroups, as such an analysis would itself introduce selection bias. Patients with confirmed active biologic therapy at the index visit were excluded from the main analysis wherever this information was available.

### Building machine learning models

The development and evaluation of predictive models followed the Transparent Reporting of a multivariable prediction model for Individual Prognosis or Diagnosis + Artificial Intelligence (TRIPOD+AI) 2024 statement. The SWMU cohort (n = 370) served as the training and internal validation set, and the Chongqing cohort (n = 180) served as the fully held-out external validation set. Continuous predictors were standardised (z-score) using means and standard deviations estimated only on the SWMU training set, and the same fitted transformation was applied unchanged to the Chongqing cohort; categorical predictors were one-hot encoded. ACQ-5, ICS dose, and log-transformed specific IgE were analyzed as continuous variables in all machine learning models, with their categorical versions retained only for descriptive tables and pre-specified sensitivity analyses (ACQ-5: <0.75/0.75–1.5/>1.5 per ATS/ERS thresholds; ICS: none/low/medium/high per GINA 2023; specific IgE: negative vs positive per ImmunoCAP grading). Missing data were <5% for all retained variables and handled by complete-case analysis. Feature selection was performed strictly inside each cross-validation fold rather than once on the full training set. Specifically, permutation importance was computed independently within each training partition of a stratified 10-fold outer loop; only variables ranking within the top five in the majority of folds were retained in the locked feature set, which was then re-fitted on the full SWMU cohort and applied unchanged to the external cohort. Hyperparameter optimisation used a nested inner 5-fold randomised search (100 iterations). Class imbalance was addressed via prevalence-inverse class weights for logistic regression (LR), Support Vector Machine with Radial Basis Function kernel (SVM-RBF), and Random Forest (RF), and via scale_pos_weight for XGBoost. For K-Nearest Neighbors (KNN), which does not accept class weights, we used distance-weighted voting (weights=‘distance’) to mitigate the influence of majority-class neighbors.

To assess the incremental value of machine learning against simpler alternatives, we compared five ML algorithms (LR, SVM-RBF, RF, XGBoost, KNN) against two pre-specified benchmark models: (i) a univariable logistic regression using peripheral blood eosinophil count alone, and (ii) a parsimonious three-variable logistic regression using age, FEV_1_%pred, and eosinophil count. The relative contribution of eosinophil indicators was further quantified by an ablation analysis sequentially removing eosinophil percentage, absolute eosinophil count, or both from the logistic-regression and XGBoost models and comparing the resulting area under the receiver operating characteristic curve (AUC) changes. Discrimination was quantified by the AUC with bootstrap 2000-resample 95% confidence intervals. Calibration was quantified by both the calibration intercept and calibration slope, in addition to graphical calibration plots. Sensitivity, specificity, positive predictive value, and negative predictive value at the Youden-optimal threshold are reported. Decision curve analysis (DCA) was performed across threshold probabilities of 0.05–0.95 to estimate net clinical benefit. To promote reproducibility, the final locked models, the preprocessing pipeline, and the analysis code will be made available upon reasonable request. Subsequently, we constructed five machine learning prediction models. These models used only peripheral blood eosinophil counts and eosinophil percentages from asthma patients. We also analyzed the calibration curves and decision curve analysis curves.

### Statistical methods and software

All analyses were performed using R software (version 4.2.1). Mendelian randomization analyses primarily used the “TwoSampleMR”, “MendelianRandomization”, and “MR-PRESSO” packages. All statistical tests were two-sided, with P < 0.05 considered statistically significant. No multiple-comparison correction was applied to descriptive baseline comparisons in **Table 1**, which are exploratory in nature and follow standard reporting conventions.

**Table 1:** Baseline characteristics by exacerbation status.

Characteristic	Overall N = 500	No N = 291	Yes N = 209	p-value
center				0.61
Chongqing	180 (32.7%)	108 (33.8%)	72 (31.3%)	
SWMU	370 (67.3%)	212 (66.2%)	158 (68.7%)	
Age	63.00 (47.00, 72.00)	64.00 (48.00, 72.00)	60.00 (46.00, 72.00)	0.31
Age (years)	63.00 (47.00, 72.00)	64.00 (48.00, 72.00)	60.00 (46.00, 72.00)	0.31
Height (cm)	166.60 (160.70, 173.60)	167.00 (160.60, 173.62)	166.50 (160.90, 173.60)	0.84
Weight (kg)	67.00 (60.62, 74.70)	67.25 (60.60, 74.93)	66.55 (60.78, 73.77)	0.37
Blood eosinophil count (×10^9^/L)	0.12 (0.05, 0.31)	0.07 (0.04, 0.15)	0.29 (0.08, 0.41)	<0.001
Eosinophil percentage (%)	1.70 (0.90, 3.08)	1.35 (0.80, 1.90)	2.90 (1.20, 3.50)	<0.001
FeNO (ppb)	38.00 (17.00, 67.00)	37.50 (17.00, 66.00)	38.00 (19.00, 71.75)	0.30
ICS dose (µg/day) ^a^	160.00 (0.00, 320.00)	160.00 (0.00, 320.00)	160.00 (160.00, 320.00)	0.024
ACQ-5 score (0-6) ^a^	2.00 (1.00, 3.00)	2.00 (1.00, 2.00)	2.00 (1.25, 3.00)	<0.001
FEV_1_ %pred (%)	87.90 (84.62, 91.80)	88.90 (85.68, 92.53)	86.55 (83.40, 90.57)	<0.001
PEF variability (%)	15.05 (9.30, 22.17)	14.10 (8.88, 21.45)	16.30 (10.15, 23.10)	0.09
FVC %pred (%)	93.20 (87.60, 97.90)	94.10 (88.30, 99.10)	92.10 (86.60, 96.38)	0.001
Total IgE (IU/mL)	32.50 (17.35, 62.05)	34.50 (18.30, 61.45)	31.90 (16.72, 62.18)	0.70
Specific IgE level (0-6) ^a^	0.00 (0.00, 0.00)	0.00 (0.00, 0.00)	0.00 (0.00, 0.00)	0.14
ICS response time (days)	0.00 (0.00, 10.40)	0.00 (0.00, 9.62)	0.00 (0.00, 11.10)	0.021
ACT score	23.00 (21.00, 25.00)	24.00 (22.00, 25.00)	23.00 (21.00, 25.00)	0.003
Gender				0.92
Female	270 (49.1%)	156 (48.8%)	114 (49.6%)	
Male	280 (50.9%)	164 (51.2%)	116 (50.4%)	
Residence				0.87
Rural	137 (24.9%)	81 (25.3%)	56 (24.3%)	
Urban	413 (75.1%)	239 (74.7%)	174 (75.7%)	
Wheezing				0.009
No	486 (88.4%)	293 (91.6%)	193 (83.9%)	
Yes	64 (11.6%)	27 (8.4%)	37 (16.1%)	

Continuous variables presented as median (Q1, Q3); categorical variables as n (%). Continuous variables compared using Wilcoxon rank-sum test; categorical variables using Pearson chi-square or Fisher’s exact test as appropriate. ^a^ACQ-5 score, ICS dose, and specific IgE level were analyzed as CONTINUOUS variables in the machine learning model.

## Result

This is an exploratory study consisting of three parts. The core focus is the role of blood eosinophils in asthma. We explicitly note that these three parts use different data sources and address distinct outcomes: (i) Mendelian randomization revealed a causal relationship between blood eosinophils and asthma in the European population; (ii) single-cell data, by comparing PBMC profiles between asthmatic and healthy controls, preliminarily uncovered the biological mechanisms of blood eosinophils in asthma; (iii) clinical data analysis and machine learning models further explored the clinical utility of blood eosinophils in asthma. The overall study design is illustrated in **Figure 1**.

**Figure 1:**
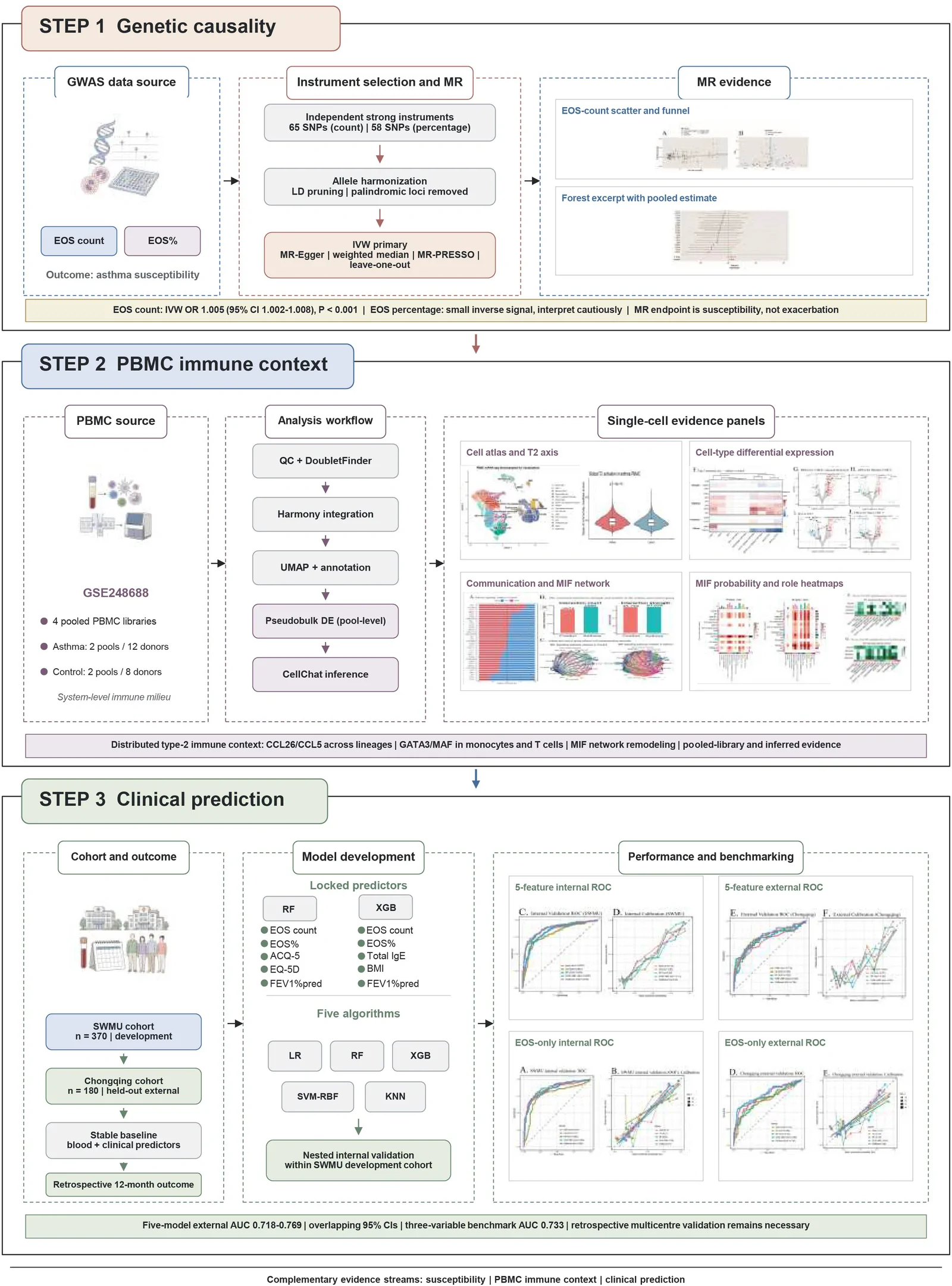
Flowchart.

### Mendelian randomization results

To investigate the causal relationship between blood eosinophil markers and asthma from a genetic perspective, this study conducted a two-sample Mendelian randomization analysis (**Table 2**). Regarding the causal relationship between absolute eosinophil count and asthma, using 65 independent SNPs as instrumental variables, the IVW estimate indicated a small but statistically significant positive association between genetically predicted eosinophil count and asthma susceptibility (OR = 1.005, 95% CI 1.002–1.008, P < 0.001) (**Figures 2A–C**). Regarding the causal relationship between eosinophil percentage and asthma, using 58 independent SNPs as instrumental variables, the IVW method revealed a negative causal association between genetically predicted eosinophil percentage and asthma risk (OR = 0.984, 95% CI: 0.970–0.998, P = 0.027) (**Figures 2D–F**). Notably, both the weighted model and the simple model reached statistical significance. However, the detailed mechanisms warrant further investigation. The MR-Egger, weighted-median, weighted-mode, and simple-mode estimators—each of which is deliberately less powerful than IVW but relaxes different assumptions about instrument validity—yielded point estimates in the same direction, although none reached nominal statistical significance individually. This pattern is consistent with the IVW signal not being predominantly driven by directional pleiotropy or by a small number of outlier instruments, but it does not constitute independent statistical replication. The MR-Egger intercept was not significantly different from zero, and MR-PRESSO detected no significant outliers. Together, these findings should be interpreted as suggestive genetic support for biological plausibility, rather than as definitive causal proof.

**Table 2:** Two-sample Mendelian randomization results for eosinophil traits and asthma risk.

Exposure	Outcome	Method	No. of SNPs	Beta (SE)	OR (95% CI)	P value	Egger intercept (SE)	P for intercept	Global.Test	Global.Test.Pvalue
EOS	Asthma	MR Egger	65	0.005 (0.003)	1.004 (0.998-1.011)	0.169	/	/	/	/
		Weighted median	65	0.003 (0.002)	1.003 (0.999-1.008)	0.102	/	/	/	/
		Inverse variance weighted	65	0.005 (0.002)	1.005 (1.002-1.008)	<0.001	/	/	/	/
		Simple mode	65	0.004 (0.004)	1.004 (0.995-1.012)	0.426	/	/	/	/
		Weighted mode	65	0.003 (0.003)	1.003 (0.998-1.008)	0.261	/	/	/	/
		Horizontal pleiotropy	/	/	/	/	4.900 (0.000)	0.855	/	/
		MR-PRESSO	/	/	/	/	/	/	84.051	0.077
EOS%	Asthma	MR Egger	58	-0.025 (0.017)	0.975 (0.944-1.008)	0.139	/	/	/	/
		Weighted median	58	-0.013 (0.009)	0.987 (0.970-0.998)	0.140	/	/	/	/
		Inverse variance weighted	58	-0.016 (0.007)	0.984 (0.970-0.998)	0.027	/	/	/	/
		Simple mode	58	-0.032 (0.014)	0.969 (0.942-0.996)	0.030	/	/	/	/
		Weighted mode	58	-0.020 (0.009)	0.980 (0.964-0.996)	0.020	/	/	/	/
		Horizontal pleiotropy	/	/	/	/	<0.001 (0.000)	0.558	/	/
		MR-PRESSO	/	/	/	/	/	/	274.782	0.282

EOS, Eosinophils; EOS%, Eosinophil Percentage; Beta represents the estimated effect (log odds) of the outcome per genetically predicted increase in exposure; OR = exp(Beta); CI, confidence interval; SE, standard error; SNP, single-nucleotide polymorphism.

**Figure 2:**
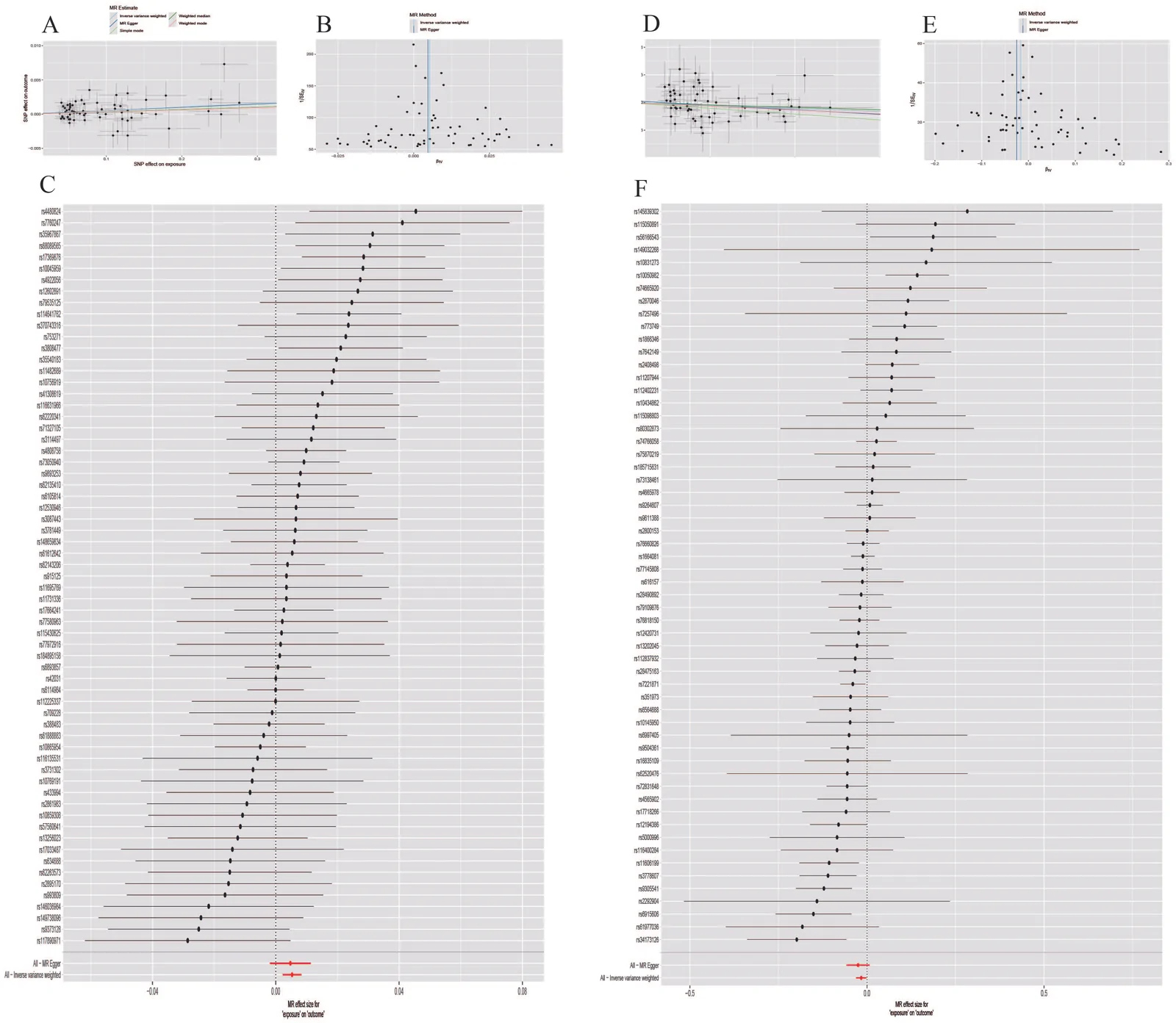
Two-sample Mendelian randomization results for eosinophil traits and asthma risk. **(A)** Scatter plot with eosinophil count as exposure; **(B)** Funnel plot with eosinophil count as exposure; **(C)** Forest plot with eosinophil count as exposure; **(D)** Scatter plot with eosinophil percentage as exposure; **(E)** Funnel plot with eosinophil percentage as exposure; **(F)** Forest plot with eosinophil percentage as exposure.

### Results of single-cell transcriptomic analysis

After stringent quality control, doublet removal by DoubletFinder, and exclusion of a manually identified B–monocyte doublet cluster, the final analysis retained a substantial number of high-quality PBMCs from four pooled samples (two asthma, two healthy control) of GSE248688. Unsupervised clustering with Harmony-corrected batch integration and marker-based annotation resolved 16 biologically distinct cell populations, including naive and memory CD4^+^ T cells, CD8^+^ T cells, NK cells, interferon-stimulated gene positive (ISG^+^) NK cells, three monocyte subsets (CD14^+^ classical, CD16^+^ non-classical, intermediate), ISG^+^ monocytes, conventional and plasmacytoid dendritic cells (pDC), conventional dendritic cell type 2 (cDC2), naive B cells, platelets, proliferating cells, HSPC, and erythrocytes (**Figure 3A**). Cell-type proportions per donor showed comparable representation of major lineages between asthma and control samples, with modest inter-sample variability in ISG^+^ monocyte and intermediate monocyte abundance (**Figure 3B**). We emphasize, as required by the study design, that these findings compare asthma patients with healthy controls and therefore reflect immune-transcriptomic features associated with asthma in general, rather than a mechanism specific to acute exacerbations. Pseudobulk differential expression analysis (DESeq2, |log2FC| > 1, padj < 0.05, blacklist-filtered) identified substantial cell-type-specific transcriptional differences between asthma and control PBMCs (**Figure 3C**). The largest number of differentially expressed genes (DEGs) was observed in CD14^+^ classical monocytes (203 up-regulated, 47 down-regulated), followed by naive CD4^+^ T cells (115 up, 137 down), memory CD4^+^ T cells (118 up, 118 down), and CD8^+^ T cells (109 up, 120 down). cDC2 (69 up, 22 down), CD16^+^ non-classical monocytes (59 up, 26 down), pDC (22 up, 12 down), and intermediate monocytes (11 up, 3 down) showed more limited but consistent transcriptional changes. This pattern indicates that both innate (monocyte/DC) and adaptive (T cell) lineages contribute to the asthma-associated transcriptional signature, with classical monocytes and CD4^+^ T cells exhibiting the most extensive remodeling. To investigate the upstream regulatory mechanisms of eosinophils in PBMCs, we evaluated a panel of eosinophil-driving axis markers. This panel included T2 cytokines (IL4, IL13), eosinophil survival factors (IL5, CSF2), eosinophil chemoattractants (CCL26, CCL5), and T2 transcription factors (GATA3, MAF). Their expression was assessed across all cell types (**Figure 3D**). Elevated expression of CCL26 and CCL5 was consistently observed across multiple asthma-derived populations (CD16^+^/CD14^+^ monocytes, naive B cells, memory CD4^+^ T, cDC2, CD8^+^ T, NK, proliferating cells, ISG^+^ NK, and platelets), whereas eosinophil-survival cytokines (IL5, CSF2) and canonical T2 cytokines (IL4, IL13) showed only weak, non-specific increases—consistent with the absence of resident eosinophils in PBMCs. Given the established molecular links between the T2 inflammation axis and eosinophil recruitment (37, 38), we computed a type-2 inflammation module score using AddModuleScore. We found that the global type-2 immunity module score was significantly higher in asthma PBMCs than in controls (Wilcoxon p < 2e−16; **Figure 3E**). To further reveal the subset distribution of T2-related gene sets (including IL4R, IL13RA1, GATA3, MAF, STAT6, JAK1, JAK2, JAK3, CCR3, AREG, CCR4, FCER1A, etc. (27912316)) in asthmatic peripheral blood, we performed a parallel T2 immune axis analysis on these genes (**Figure 3F**). The result demonstrated that GATA3 and MAF were significantly up-regulated in CD14^+^ classical monocytes (*** p < 0.001), CD16^+^ non-classical monocytes (* p < 0.05), naive CD4^+^ T cells (* p < 0.05), memory CD4^+^ T cells (* p < 0.05), and CD8^+^ T cells (** p < 0.01). JAK1 and JAK3 signaling components were selectively up-regulated in CD8^+^ T cells and memory CD4^+^ T cells. In contrast, T2 receptors (IL4R, IL13RA1, IL5RA) showed limited change, and FCER1A was significantly down-regulated in monocyte and T cell subsets, consistent with a partial redistribution of high-affinity IgE receptor expression under sustained T2 exposure. Together, these results suggest that systemic T2 transcriptional priming in asthma is distributed across multiple immune lineages—particularly monocytes and CD4^+^ T cells—rather than being confined to a single “effector” population.

**Figure 3:**
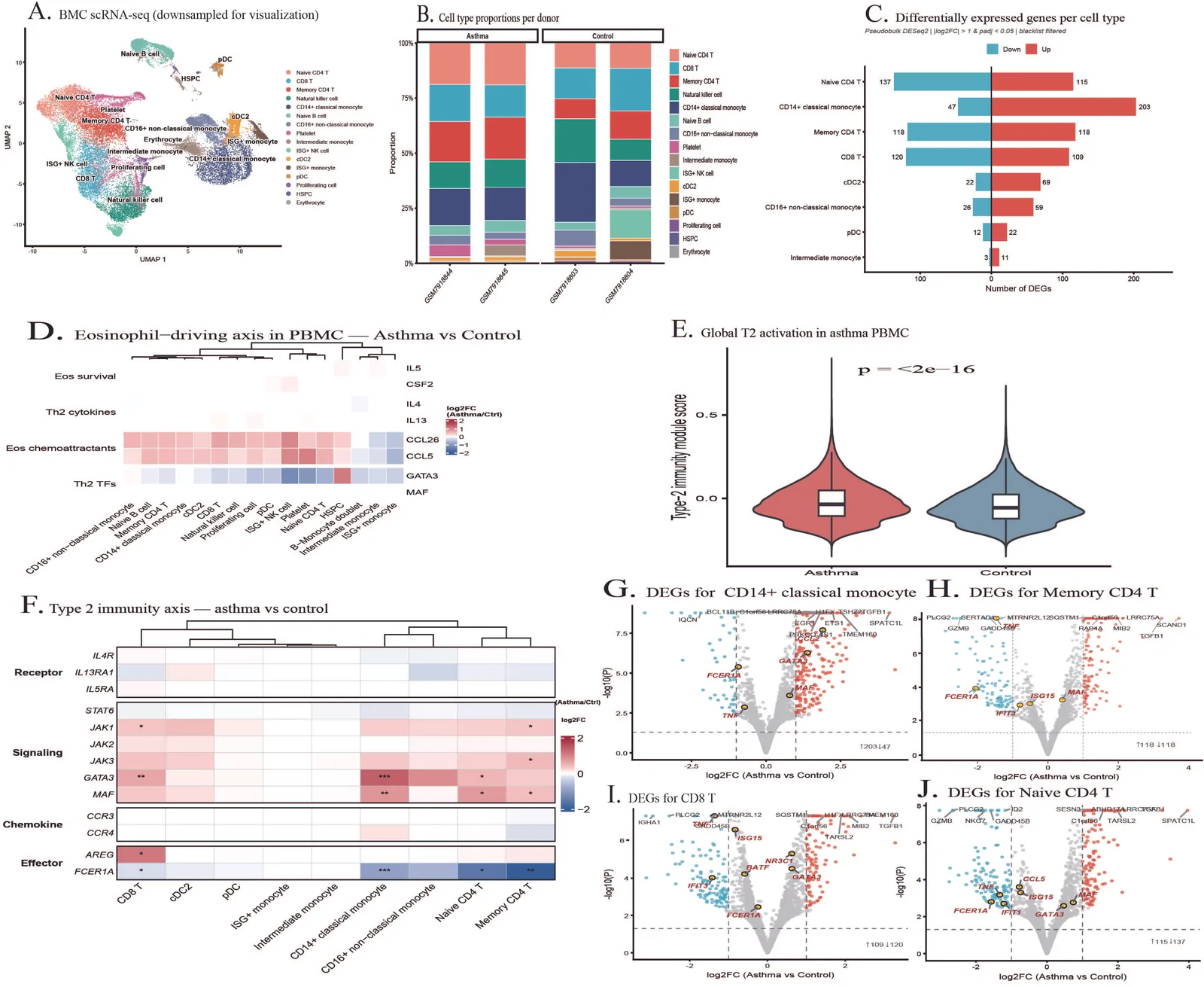
Single-cell transcriptomic landscape and type-2 immune priming in asthma versus healthy PBMCs. **(A)** UMAP visualization of PBMCs from four pooled samples (two asthma, two healthy control) after quality control, DoubletFinder-based doublet removal, Harmony batch correction, and marker-based annotation; **(B)** Stacked bar plot showing cell-type proportions per donor (GSM7918844 and GSM7918845, asthma; GSM7918803 and GSM7918804, control); **(C)** Number of differentially expressed genes (DEGs) per cell type identified by pseudobulk DESeq2 analysis (asthma vs control; |log_2_FC| > 1, adjusted p < 0.05; filtered against a three-tier blacklist covering structural transcripts, platelet-derived ambient RNA, and myeloid-derived ambient RNA); **(D)** Heatmap of log_2_ fold changes (asthma/control) for the eosinophil-driving axis; **(E)** Violin plot of the global type-2 immunity module score (Seurat AddModuleScore) at the single-cell level in asthma versus control PBMCs; **(F)** Heatmap of log_2_ fold changes (asthma/control) for the Type 2 immunity axis; **(G–J)** Volcano plots of DEGs (pseudobulk DESeq2) in **(G)** CD14^+^ classical monocytes, **(H)** memory CD4^+^ T cells, **(I)** CD8^+^ T cells, and **(J)** naive CD4^+^ T cells. * p < 0.05; ** p < 0.01; *** p < 0.001.

To infer intercellular communication changes associated with asthma, CellChat was applied to the annotated dataset. The total number of inferred ligand–receptor interactions was higher in asthma (n = 2,866) than in control (n = 2,574), with modestly increased overall interaction strength (**Figure 4B**). Pathway-level information-flow analysis (**Figure 4A**) revealed that several immune signaling pathways were more active in asthma, most prominently MIF (log2FC ≈ 1.29, information flow 7.38 → 18.04), along with ALCAM/CD6, FLT3, APP, CD86, ADGRE5, SELL, CLEC, CD22, and CD45. Conversely, information flow through CXCL, BAG, SN, GRN, JAM, VISFATIN, SEMA4, BAFF, ICAM, ANNEXIN, and ITGB2 was reduced in asthma. These shifts are consistent with a rebalancing of chemokine-driven and adhesion-driven communication toward MIF-, co-stimulation-, and lectin-mediated interactions in the asthmatic circulation. Because MIF exhibited the largest positive shift among pathways with substantial baseline flow, we examined it in detail. Comparative network diagrams (**Figure 4C**) and communication-probability heatmaps (**Figures 4D, E**) showed that MIF signaling in the control state was concentrated among a restricted set of sender–receiver pairs, whereas in asthma the network became broader and more densely connected, with CD14^+^ classical monocytes, CD16^+^ non-classical monocytes, intermediate monocytes, cDC2, and pDC serving as prominent MIF senders, and lymphoid populations (naive/memory CD4^+^ T, CD8^+^ T, NK, naive B cells) acting as principal receivers. Sender/receiver/mediator/influencer role analysis (**Figures 4F, G**) further suggested that monocyte and DC subsets shifted toward stronger sender and influencer roles in asthma, whereas T-cell subsets became more prominent receivers and mediators. It is important to note that these CellChat inferences reflect coordinated ligand–receptor gene co-expression, and therefore should be interpreted as hypothesis-generating patterns of possible communication rather than as direct experimental evidence of functional signaling.

**Figure 4:**
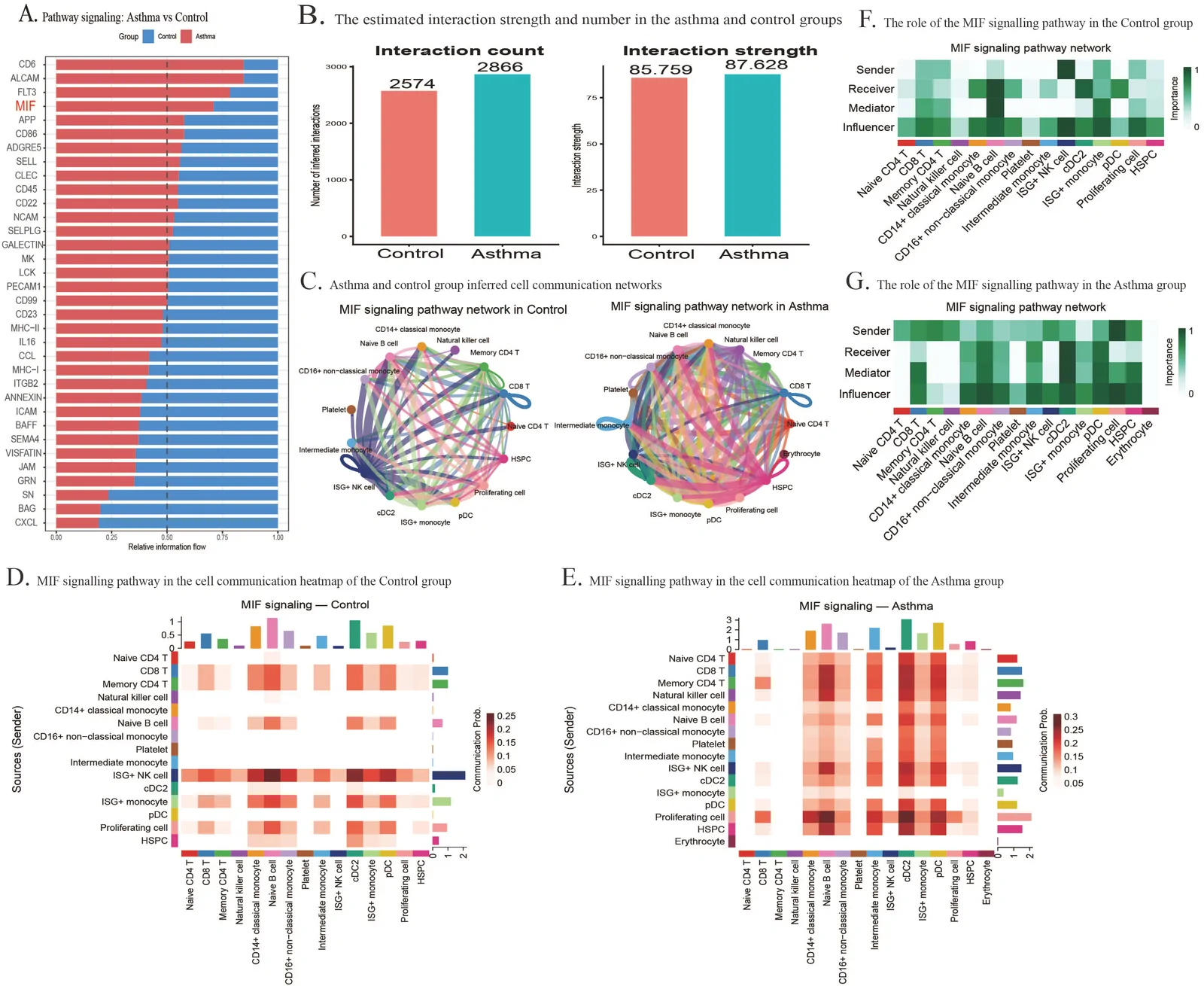
CellChat-inferred intercellular communication landscape and MIF signaling remodeling in asthma versus healthy PBMCs. **(A)** Ranked comparison of relative information flow for all signaling pathways detected by CellChat between asthma and control PBMCs; **(B)** Total number (left) and total interaction strength (right) of inferred ligand–receptor communications between asthma and control groups; **(C)** Cell–cell communication network diagrams of the MIF signaling pathway in the control (left) and asthma (right) groups; **(D, E)** Heatmaps of MIF-pathway communication probability between sender (rows) and receiver (columns) cell populations in **(D)** the control group and **(E)** the asthma group; **(F, G)** Sender/receiver/mediator/influencer role heatmaps for the MIF signaling pathway in **(F)** the control group and **(G)** the asthma group.

### Results of clinical data statistical analysis

During the 12-month retrospective follow-up, 230 of 550 patients (41.8%) experienced at least one ATS/ERS-defined asthma exacerbation (exacerbation group), while 320 patients (58.2%) remained exacerbation-free (non-exacerbation group). Baseline demographic and clinical characteristics of the two groups are summarized in **Table 1**. Demographic characteristics were balanced between groups. Age (median 60 vs 64 years, P = 0.31), sex distribution (male 50.4% vs 51.2%, P = 0.92), height (P = 0.84), weight (P = 0.37), residence (urban 75.7% vs 74.7%, P = 0.87), and study-center distribution (P = 0.61) did not differ significantly, indicating that the two groups were demographically comparable and that the observed differences in downstream clinical variables are unlikely to be confounded by basic demographic factors. Eosinophil-related indicators showed the most striking between-group differences. The absolute blood eosinophil count was markedly higher in exacerbators than in non-exacerbators [0.29 (0.08, 0.41) × 10^9^/L vs 0.07 (0.04, 0.15) × 10^9^/L, P < 0.001], with the median in the exacerbation group being approximately 4-fold that of the non-exacerbation group. Similarly, the eosinophil percentage was significantly elevated in the exacerbation group [2.90% (1.20%, 3.50%) vs 1.35% (0.80%, 1.90%), P < 0.001], with an approximately 2-fold difference in medians. These findings identify peripheral blood eosinophil metrics as the two clinical variables with the strongest univariable association with exacerbation risk in this cohort, providing preliminary support for their use as predictive biomarkers. Airflow and asthma-control indicators were also significantly worse in exacerbators. FEV_1_%pred was lower in the exacerbation group [86.55% (83.40, 90.57) vs 88.90% (85.68, 92.53), P < 0.001], as was FVC%pred [92.10% (86.60, 96.38) vs 94.10% (88.30, 99.10), P = 0.001]. PEF variability trended higher in exacerbators [16.30% (10.15, 23.10) vs 14.10% (8.88, 21.45), P = 0.09] but did not reach conventional significance. Patients in the exacerbation group had higher ACQ-5 scores [2.00 (1.25, 3.00) vs 2.00 (1.00, 2.00), P < 0.001], lower ACT scores [23 (21, 25) vs 24 (22, 25), P = 0.003], and lower EQ-5D health-utility values [0.97 (0.88, 1.00) vs 1.00 (0.93, 1.00), P = 0.002], indicating poorer symptom control and health-related quality of life at baseline. Treatment- and clinical-course variables also differed. Baseline ICS daily dose was slightly higher in the exacerbation group [160 (160, 320) vs 160 (0, 320) µg/day, P = 0.024], and ICS response time was longer [median 0.00 days but higher upper quartile: 11.10 vs 9.62 days, P = 0.021], suggesting subtly greater treatment intensity and delayed corticosteroid responsiveness among patients who subsequently exacerbated. The prevalence of wheezing was higher in the exacerbation group (16.1% vs 8.4%, P = 0.009). In contrast, several variables did not differ significantly between groups, including FeNO (38 vs 37.5 ppb, P = 0.30), total IgE (31.9 vs 34.5 IU/mL, P = 0.70), specific IgE level (P = 0.14), allergic rhinitis prevalence (50.0% vs 51.2%, P = 0.84), and bronchodilator-test positivity (0.9% vs 0%, P = 0.17). The lack of differences in FeNO, total IgE, and specific IgE—despite the strong differences in eosinophil metrics—suggests that peripheral blood eosinophil measurement captures information about exacerbation risk that is not redundant with airway type-2 inflammation (FeNO) or systemic atopy (IgE) in this cohort.

The final analytic cohort comprised 550 adult asthma patients from two clinical centers: 370 patients (67.3%) from the Affiliated Hospital of Southwest Medical University and 180 patients (32.7%) from the People’s Hospital of Shapingba District, Chongqing. Baseline demographic and clinical characteristics of center are summarized in **Supplementary Table 1**. To assess the comparability of the two centers, we compared all continuous and categorical baseline variables between SWMU and Chongqing (**Supplementary Table 1**). Age (median 62.5 vs 64.5 years; P = 0.205), sex (male 49.2% vs 54.4%; P = 0.286), BMI (24.04 vs 24.37 kg/m²; P = 0.425), and residence distribution (P = 0.234) were comparable. Critically, the two key eosinophil indicators showed near-identical distributions across centers — absolute eosinophil count (median 0.12 × 10^9^/L in both centers; P = 0.983) and eosinophil percentage (1.70% in both; P = 0.507) — as did FeNO (P = 0.411), FEV_1_%pred (P = 0.075), FVC%pred (P = 0.377), PEF variability (P = 0.386), total IgE (P = 0.570), ACT (P = 0.427), ACQ-5 (P = 0.086), EQ-5D index (P = 0.597), ICS daily dose (P = 0.352), ICS response time (P = 0.284), and prior exacerbation frequency (P = 0.610). Categorical variables including wheezing (P = 0.197), allergic rhinitis (P = 1.000), bronchodilator-test positivity (P = 1.000), ACQ-5 category (P = 0.182), ICS dose category (P = 0.369), and specific IgE category (P = 0.587) were also balanced. These results demonstrate that the two cohorts constitute a homogeneous population, thereby supporting the SWMU cohort as the training set and the Chongqing cohort as an appropriate independent external validation set. Furthermore, baseline characteristics were compared between the exacerbation and non-exacerbation groups within each center (**Supplementary Table 2**) to verify the reproducibility of the association between eosinophil markers and asthma exacerbations.

### Results of machine learning predictive models

Feature selection was performed strictly inside each training fold of the stratified 10-fold cross-validation using permutation importance, thereby preventing information leakage from the external validation set. Across folds, five variables consistently ranked in the top five: absolute eosinophil count (EOS ×10^9^/L), eosinophil percentage, ACQ-5 score, EQ-5D health utility index, and FEV_1_%pred (**Figures 5A, B**). Both Random Forest and XGBoost feature-importance rankings independently placed absolute eosinophil count and eosinophil percentage at the top two positions, with importance values substantially exceeding those of remaining features. This locked five-variable feature set was then re-trained on the full SWMU cohort and applied unchanged to the Chongqing external validation cohort. Three variables were treated as continuous in all machine learning models: ACQ-5 score (0–6), ICS daily dose (µg/day, budesonide-equivalent), and specific IgE level (CAP class 0–6), in accordance with their original scoring conventions. Pre-specified sensitivity analyses using clinically accepted thresholds (ACQ-5: <0.75/0.75–1.5/>1.5 per ATS/ERS; ICS: none/low/medium/high per GINA 2023; specific IgE: negative vs positive per CAP grading) are reported in **Supplementary Table 3** (categorical version, descriptive only) and did not materially change the primary conclusions.

**Figure 5:**
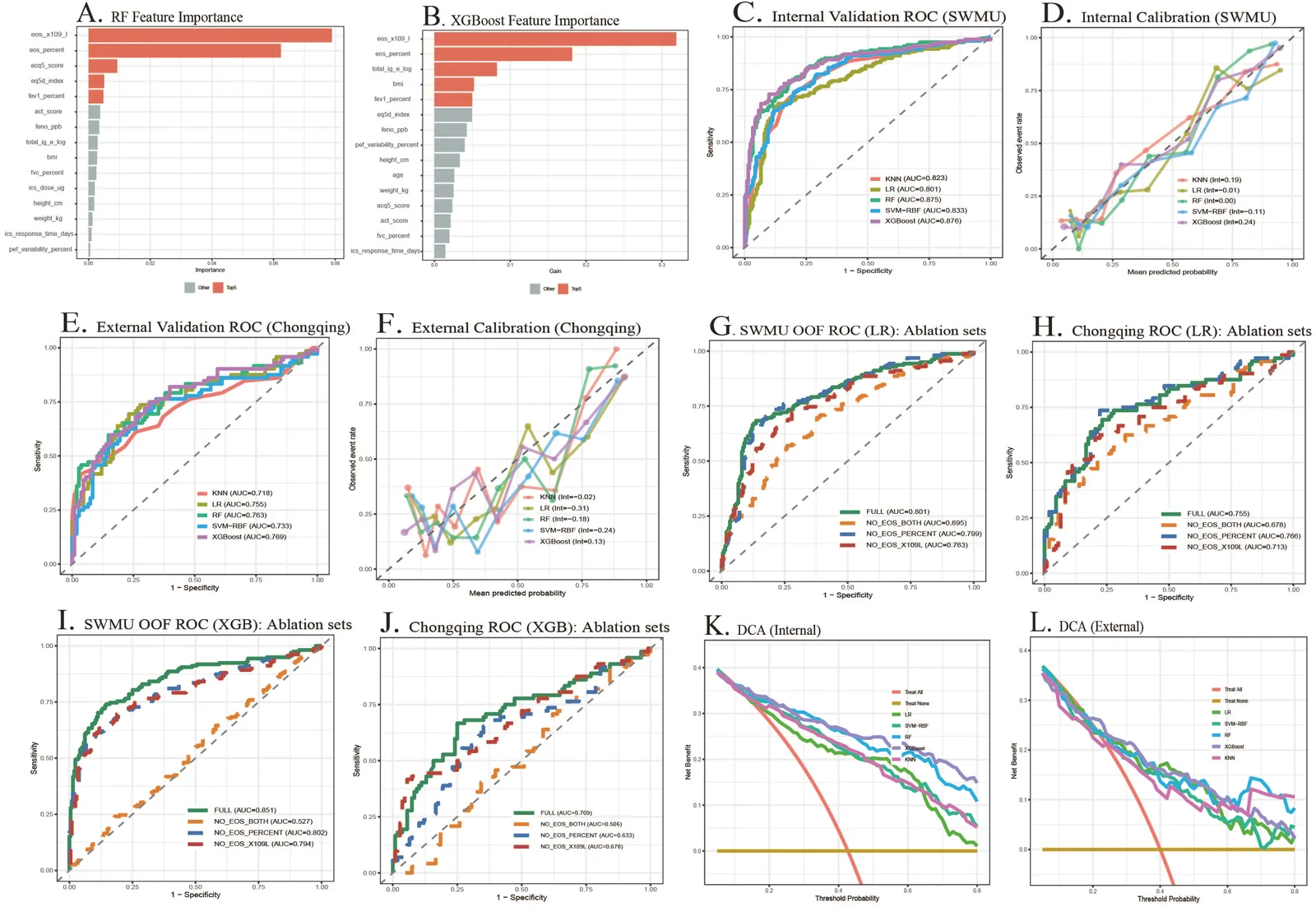
Development and validation analysis of the five-feature machine learning model for predicting asthma exacerbation. **(A)** Permutation-based feature importance (mean decrease in accuracy) for Random Forest; **(B)** gain-based feature importance for XGBoost; **(C)** Internal-validation ROC on the SWMU training cohort (n = 370); **(D)** Internal-calibration plot on the SWMU cohort; **(E)** External-validation ROC on the Chongqing cohort (n = 180); **(G–J)** Ablation analysis quantifying the marginal contribution of the two eosinophil indicators. ROC curves comparing the full five-feature model (FULL) with three ablated variants: NO_EOS_PERCENT (eosinophil percentage removed), NO_EOS_X10^9^L (absolute eosinophil count removed), and NO_EOS_BOTH (both eosinophil indicators removed). Panels G and H show LR results on the SWMU internal and Chongqing external cohorts, respectively; panels I and J show the corresponding XGBoost results; **(K, L)** Decision-curve analysis demonstrating clinical utility. Net benefit as a function of decision threshold probability for all five models on the SWMU internal cohort **(K)** and the Chongqing external cohort **(L)**. ACQ-5, 5-item Asthma Control Questionnaire; AUC, area under the receiver-operating-characteristic curve; DCA, decision-curve analysis; EOS, eosinophil; EQ-5D, EuroQol 5-Dimension health-utility index; FEV_1_%pred, forced expiratory volume in 1 second, percent predicted; FVC, forced vital capacity; FeNO, fractional exhaled nitric oxide; ICS, inhaled corticosteroid; Int, calibration intercept; KNN, k-nearest neighbours; LR, logistic regression; OOF, out-of-fold; PEF, peak expiratory flow; RF, random forest; ROC, receiver operating characteristic; SVM-RBF, support vector machine with radial-basis-function kernel; SWMU, Southwest Medical University; XGB/XGBoost, extreme gradient boosting.

Internal validation on the SWMU cohort using nested 10-fold cross-validation yielded the following AUCs with bootstrap 2000-resample 95% confidence intervals (**Figure 5C**): XGBoost 0.876 (95% CI 0.836–0.913), RF 0.875 (95% CI 0.834–0.912), SVM-RBF 0.833 (95% CI 0.787–0.876), KNN 0.823 (95% CI 0.774–0.869), and LR 0.801 (95% CI 0.751–0.850). Because the 95% confidence intervals of the top-performing algorithms substantially overlap, we refrain from claiming that any single algorithm is definitively superior. Calibration analysis (**Figure 5D**) showed that LR and RF had near-ideal calibration intercepts (LR = −0.01; RF = 0.00), whereas XGBoost (Int = 0.24) and KNN (Int = 0.19) exhibited mild systematic over-prediction, and SVM-RBF (Int = −0.11) showed slight under-prediction. External validation on the fully held-out Chongqing cohort—applied without any re-tuning or refitting of the preprocessing pipeline—yielded expectedly lower but clinically meaningful AUCs (**Figure 5E**): XGBoost 0.769 (95% CI 0.696–0.837), RF 0.763 (95% CI 0.690–0.831), LR 0.755 (95% CI 0.680–0.824), SVM-RBF 0.733 (95% CI 0.656–0.804), and KNN 0.718 (95% CI 0.639–0.791). External calibration (**Figure 5F**) revealed systematic under-prediction across most models (LR Int = −0.31; SVM-RBF Int = −0.24; RF Int = −0.18; KNN Int = −0.02), whereas XGBoost showed mild over-prediction (Int = 0.13). Sensitivity, specificity, PPV, and NPV at Youden-optimal thresholds for each model, along with the two benchmark comparators, are reported in **Supplementary Table 4**. Compared with an eosinophil-count-only univariable logistic-regression benchmark (external AUC 0.680, 95% CI 0.596–0.765) and a parsimonious three-variable clinical model (age + FEV_1_%pred + eosinophil count; external AUC 0.733, 95% CI 0.657–0.810), the five-feature machine learning models provided a moderate incremental discriminative gain of approximately 0.03–0.09 in AUC. Ablation analysis was performed to quantify the marginal contribution of the two eosinophil indicators using LR and XGBoost as representatives (**Figures 5G–J**). In the LR model on the SWMU internal set (**Figure 5G**), the full model achieved AUC = 0.801, which decreased marginally to 0.799 after removing eosinophil percentage (Δ = −0.002), decreased to 0.763 after removing absolute eosinophil count (Δ = −0.038), and dropped substantially to 0.695 after removing both eosinophil indicators (Δ = −0.106). On the Chongqing external set (**Figure 5H**), the corresponding LR AUCs were 0.755 (full), 0.766 (no percent, Δ = +0.011), 0.713 (no absolute, Δ = −0.042), and 0.678 (no both, Δ = −0.077). In XGBoost (**Figures 5I, J**), the ablation effect was more pronounced: on the SWMU set, the AUC dropped from 0.851 (full) to 0.527 (no both, Δ = −0.324); on the Chongqing external set, from 0.709 (full) to 0.506 (no both, Δ = −0.203). We note that removing eosinophil percentage alone produced negligible or even trivially positive AUC changes in LR, indicating that the two eosinophil indicators are highly collinear and that most of their joint predictive information can be captured by absolute eosinophil count alone in a linear model. In the tree-based XGBoost model, however, both indicators contributed measurable non-redundant signal. Decision curve analysis (**Figures 5K, L**) confirmed that all five machine learning models provided positive net clinical benefit compared with “treat-all” and “treat-none” strategies across a wide range of threshold probabilities (~0.2–0.8) in both the internal SWMU cohort and the external Chongqing cohort, with the net-benefit curves of the top-performing models (RF, XGBoost, LR) tightly overlapping.

To evaluate whether peripheral blood eosinophils alone—the two most accessible and lowest-cost clinical biomarkers in this study—can provide clinically useful prediction of 12-month exacerbation risk, we developed a parallel set of five machine learning models using only two features: absolute eosinophil count (EOS ×10^9^/L) and eosinophil percentage (EOS%). All other elements of the analytical pipeline (nested cross-validation on the SWMU training cohort, hyperparameter tuning, probability calibration, and full external validation on the independent Chongqing cohort) were held identical to those of the five-feature model in **Figure 5**, so that the two figures are directly comparable and jointly quantify the marginal contribution of the non-eosinophil clinical variables. Discrimination on the SWMU internal cohort (**Figure 6A**) yielded the following out-of-fold AUCs with bootstrap 2000-resample 95% confidence intervals: RF 0.865 (95% CI 0.824–0.902), XGBoost 0.863 (95% CI 0.822–0.901), SVM-RBF 0.846 (95% CI 0.802–0.886), KNN 0.841 (95% CI 0.795–0.883), and LR 0.771 (95% CI 0.719–0.821). Notably, the tree-based ensembles (RF, XGBoost) and the non-linear SVM-RBF achieved AUCs that were essentially indistinguishable from those of the corresponding five-feature models in **Figure 5** (0.875, 0.876, and 0.833 respectively). Calibration on the SWMU internal cohort (**Figure 6B**) was excellent for all models, with calibration intercepts close to zero (LR = −0.01, RF = 0.00, SVM-RBF = 0.00, XGBoost = 0.01, KNN = 0.10), indicating good agreement between predicted probabilities and observed event rates in the internal setting. Decision curve analysis (**Figure 6C**) showed that all five eosinophil-only models delivered positive net clinical benefit over “treat-all” and “treat-none” strategies across a wide range of threshold probabilities (~0.15–0.60), with the top four algorithms (RF, XGBoost, SVM-RBF, KNN) showing tightly overlapping curves. This observation should be interpreted cautiously given overlapping confidence intervals.

**Figure 6:**
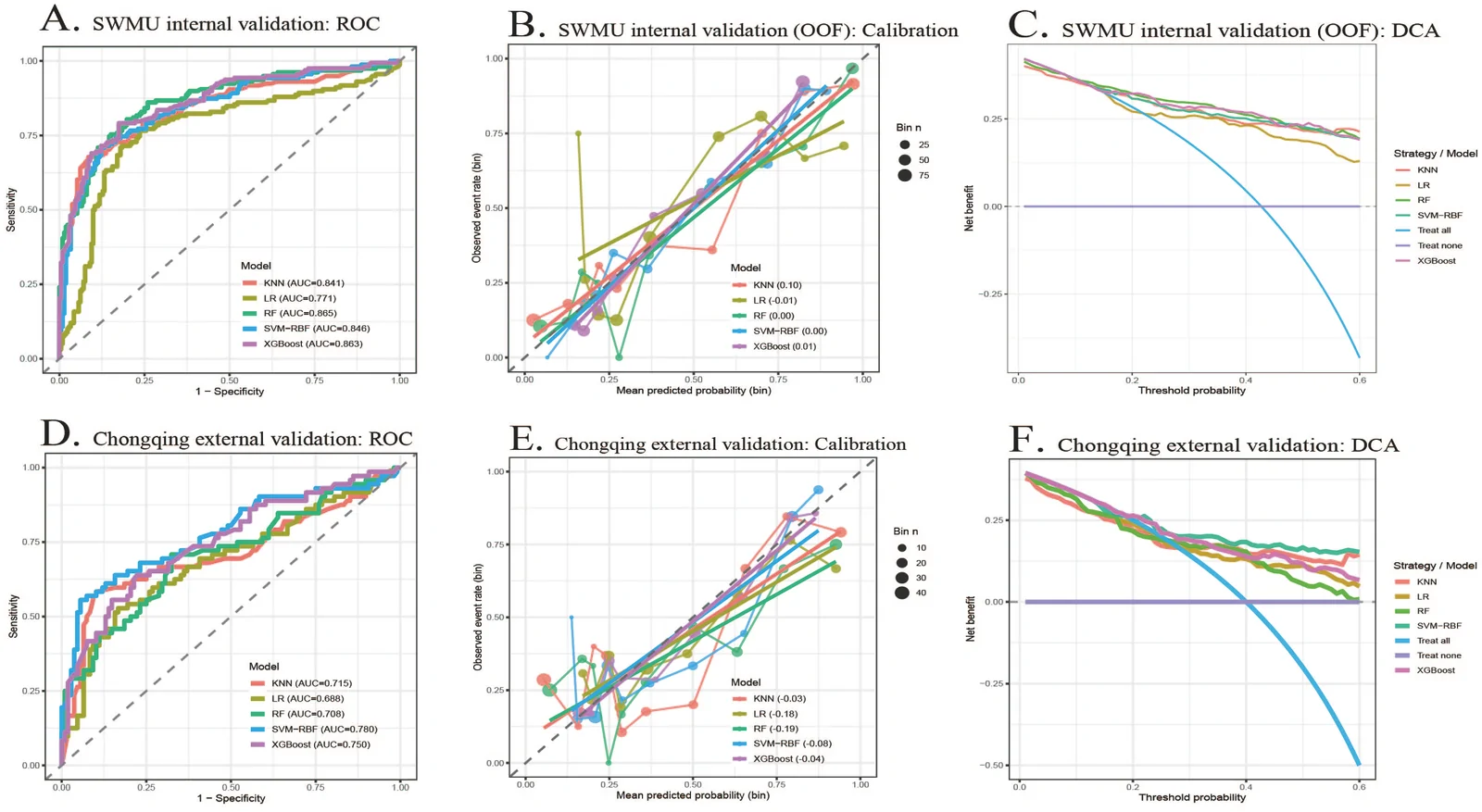
Performance of an eosinophil-only two-feature machine learning model for predicting asthma exacerbation. **(A)** SWMU internal-validation ROC; **(B)** SWMU internal-validation calibration (OOF); **(C)** SWMU internal-validation decision-curve analysis (OOF); **(D)** Chongqing external-validation ROC; **(E)** Chongqing external-validation calibration; **(F)** Chongqing external-validation decision-curve analysis. AUC, area under the receiver-operating-characteristic curve; DCA, decision-curve analysis; EOS, eosinophil; KNN, k-nearest neighbours; LR, logistic regression; OOF, out-of-fold; RF, random forest; ROC, receiver operating characteristic; SVM-RBF, support vector machine with radial-basis-function kernel; SWMU, Southwest Medical University; XGBoost, extreme gradient boosting.

## Discussion

For a long time, eosinophil recruitment has been regarded as a hallmark of the inflammatory response in asthma. Eosinophils are considered effector cells in asthma, but little is known about the functional significance of their precise localization within the airway wall. Both intraepithelial and subepithelial eosinophils are associated with type 2 inflammation, with the strongest association observed between IL-5 expression and intraepithelial eosinophils (39). Furthermore, eosinophils cause tissue damage through eosinophil-derived cytotoxic mediators, which contribute to eosinophilic airway inflammation and drive the onset and progression of asthma (40). Accordingly, peripheral blood eosinophils in asthma patients are thought to be of potential clinical relevance. In this three-component exploratory study, we investigated whether blood eosinophil traits carry causal, mechanistic, and clinical signals relevant to asthma in adults. Two-sample Mendelian randomization supported a modest but robust causal effect of genetically predicted absolute eosinophil count on asthma susceptibility (IVW OR 1.005, 95% CI 1.002–1.008), whereas single-cell transcriptomics of peripheral blood mononuclear cells revealed a systemic type-2 transcriptional programme distributed across monocytes and CD4^+^ T cells rather than confined to eosinophils themselves. Clinically, five machine learning models trained on the SWMU cohort achieved external AUCs of 0.718–0.769 in the Chongqing cohort, with substantially overlapping confidence intervals and only modest gains over a three-variable benchmark model (AUC 0.733). Taken together, these findings position blood eosinophil count as a biologically plausible but incrementally modest predictor of 12-month exacerbations, rather than a stand-alone clinical decision tool.

The positive causal effect of absolute eosinophil count on asthma susceptibility is directionally consistent with the well-established role of eosinophilic inflammation in type-2-high asthma (37, 38) and with meta-analytic evidence that elevated blood eosinophils are associated with exacerbations (25). A landmark UK Biobank Mendelian randomization study by Zhu et al. (41) similarly reported that genetically instrumented eosinophil counts increase the odds of adult-onset asthma (OR ≈ 1.20 per SD), and Ferreira et al. (42) identified 99 asthma-associated loci with strong colocalization to eosinophil-count trait signals in a Nature Genetics genome-wide association study. Our IVW estimate (OR 1.005 per unit) is numerically smaller because the exposure was scaled per raw count rather than per SD, but the direction and robustness across MR-Egger, weighted median, and MR-PRESSO analyses are fully concordant with these prior reports. However, the MR outcome (asthma diagnosis) is not identical to the clinical prediction endpoint (prospective exacerbation), and this analysis should therefore be interpreted as evidence of biological plausibility rather than as validation of the downstream model. The apparent negative association for eosinophil percentage (IVW OR 0.984, 95% CI 0.970–0.998) is small, borderline, and directionally inconsistent with our clinical data, in which exacerbators had higher eosinophil percentages. We interpret this as most plausibly reflecting the compositional nature of a percentage measurement: a rise in eosinophil percentage can result from an absolute eosinophil increase, from a fall in other leukocyte populations, or from correlated genetic instruments acting on total white-cell composition (43). We therefore now present the percentage result as an unexpected, hypothesis-generating finding requiring adjustment for neutrophil and total leukocyte counts in future work, rather than as evidence of a protective effect.

Because mature eosinophils are largely lost during Ficoll separation of PBMCs (12), and because GSE248688 contrasts asthma patients with healthy controls rather than exacerbating with stable asthma, our scRNA-seq analysis is best interpreted as characterising the upstream immune milieu that supports peripheral eosinophil biology in asthma in general. Consistent with this reframing, the largest transcriptional remodelling occurred in CD14^+^ classical monocytes (203 up-regulated genes) and CD4^+^ T-cell subsets, with elevated CCL26 and CCL5 across multiple lineages and up-regulated GATA3/MAF in monocytes and T cells—together suggesting a distributed rather than eosinophil-restricted type-2 programme (44, 45). These observations converge on emerging concepts of a distributed rather than lymphocyte-restricted type-2 immune programme in asthma. Nakayama et al. (44) and Fahy (37) have argued that GATA3-driven T2 signatures are increasingly detected outside canonical Th2 cells, including in innate lymphoid cell 2 (ILC2) populations and myeloid subsets. Our finding of GATA3/MAF up-regulation in CD14^+^ monocytes are consistent with the report by Kim et al. (46) that monocyte-derived cells acquire a GATA3^+^ IL-13-producing phenotype under sustained IL-4/IL-13 exposure, and with Lambrecht & Hammad’s (38) paradigm that airway and blood monocytes function as amplifiers—not mere responders—of type-2 inflammation. The elevated CCL26 expression we observed in platelet and monocyte clusters is particularly noteworthy, as CCL26 (eotaxin-3) has been identified by Larose et al. (47) as the dominant eosinophil chemoattractant in the airway epithelium of T2-high asthma and correlates with IL-13 signalling; its presence in circulating myeloid and platelet populations suggests that peripheral eosinophil recruitment may be primed before eosinophils reach the airway.

The prominence of MIF (macrophage migration inhibitory factor) signalling in our CellChat analysis (information flow 7.38 → 18.04; log_2_FC ≈ 1.29) is a novel finding that aligns with, but extends, prior murine and human data. These findings suggest that peripheral eosinophil enrichment in asthma may be sustained through at least three non-exclusive routes: bone-marrow release (HSPC signalling), survival support in peripheral blood (CCL26/CCL5 from monocytes and platelets), and peripheral T2 priming (GATA3/MAF activation). Mizue et al. (48) showed that MIF is markedly elevated in asthmatic airways and that MIF-knockout mice are protected from allergen-induced eosinophilic inflammation. Bozza et al. (49) further demonstrated that MIF stabilises eosinophil chemotaxis and prolongs eosinophil survival via CD74/CXCR4 signalling. Our observation that CD14^+^ monocytes, platelets, and HSPCs jointly act as MIF senders in asthma PBMCs suggests a tripartite peripheral MIF axis (marrow release → circulating survival support → myeloid activation) that may sustain the peripheral eosinophil pool. Nevertheless, CellChat and UMAP-based analyses infer, rather than prove, functional communication (50).

We built predictive models based on clinical features, especially peripheral blood eosinophil indices in asthma patients. We then report a head-to-head comparison against two pre-specified benchmark models. In external validation, our five-feature machine learning models achieved AUCs of 0.718–0.769, compared with 0.680 (95% CI 0.596–0.765) for eosinophil count alone and 0.733 (0.657–0.810) for the three-variable clinical model (age + FEV_1_ %pred + eosinophil count). The incremental gain over the parsimonious benchmark was modest (ΔAUC ≈ −0.015 to +0.037), and 95% confidence intervals substantially overlapped across all five algorithms. We therefore do not claim that any single algorithm is definitively superior. This modest incremental value of complex ML over parsimonious clinical models mirrors findings across the broader asthma prediction literature. Loymans et al. (51) developed and externally validated a clinical prediction rule for severe exacerbations in 611 primary-care patients, achieving an AUC of 0.71 using only routinely collected variables (previous exacerbations, ACQ, spirometry)—almost identical to our benchmark model. More recently, Tong et al. (52) applied XGBoost to 60,302 Medicare beneficiaries and reported an AUC of 0.75 for 12-month exacerbation, again with only marginal gains over logistic regression. The convergence of external AUCs around 0.70–0.77 across geographically and technically diverse cohorts suggests a soft ceiling on exacerbation prediction using routine clinical variables, likely driven by the multifactorial and partly stochastic nature of exacerbation triggers (viral infection, allergen exposure, adherence lapses) (53) rather than by any single algorithmic limitation. The comparability of eosinophil-based prediction to established composite scores is also informative. Price et al. (54), in a 130,547-patient UK primary-care study, showed that blood eosinophil count ≥ 400 cells/μL alone predicted asthma exacerbations with an incidence rate ratio of 1.42 (95% CI 1.35–1.49). Our univariable eosinophil-only benchmark (external AUC 0.680) is consistent with this magnitude and confirms that eosinophil count carries substantial standalone prognostic information, though it captures only part of the risk profile. At Youden-optimal thresholds, XGBoost achieved 60% sensitivity and 84% specificity externally, compared to 56% sensitivity/85% specificity for the three-variable benchmark, indicating clinically similar operating characteristics.

Admittedly, although our study can preliminarily observe the role of peripheral blood eosinophils in asthma patients, it still has several limitations. First, because this study employed three methods (i.e., Mendelian randomization, single-cell analysis, and clinical data-based predictive model construction), the data for these three approaches correspond to different populations. In addition, the external validation cohort remains modest in size (n = 180) and originates from the same broader healthcare setting as the derivation cohort, so generalizability to other ethnic populations, pediatric asthma, and primary-care settings requires further evaluation. Therefore, our results only suggest a trend or direction, and further larger-scale prospective studies are needed to confirm the role of peripheral blood eosinophils in asthma, after adjusting for confounding factors such as population differences. Second, retrospective extraction of biologic-therapy status and detailed prior-exacerbation history was incomplete, and residual confounding by disease severity, adherence, and unmeasured infections cannot be excluded (51). Third, the MR analysis addresses asthma susceptibility rather than exacerbation risk, and the scRNA-seq dataset compares asthma with healthy controls rather than exacerbating with stable asthma; the three components therefore provide complementary rather than confirmatory evidence. Fourth, GSE248688 provided only four pooled samples without patient-level demultiplexing, limiting the statistical unit to pseudo-replicates rather than donors (55). Fifth, we did not incorporate longitudinal eosinophil trajectories, which recent work by Kerkhof et al. (56) suggests may carry stronger prognostic information than single-point measurements. Finally, the present study concerns adult asthma only; extrapolation to pediatric populations, in whom viral triggers dominate and blood eosinophil kinetics differ is not supported by our data (57, 58).

## Conclusion

Our study supports peripheral blood eosinophil counts in adult asthma patients as a biologically plausible and clinically relevant potential predictor. A simple three-variable model performed comparably to more complex machine learning approaches, arguing for parsimony in clinical translation. Prospective multi-center validation, incorporation of longitudinal eosinophil trajectories, and integration with FeNO and biologic-therapy status will be needed before eosinophil-based prediction can be considered ready for clinical implementation.

## Data Availability

The datasets presented in this study can be found in online repositories. The names of the repository/repositories and accession number(s) can be found in the article/**Supplementary Material**.
