# Supplementary material for: Blood eosinophils as predictors of asthma exacerbations in adults: converging evidence from mendelian randomization, single-cell transcriptomics, and a two-center longitudinal cohort
Source: Front Immunol. 2026 Sep 22;17:1927894. doi: 10.3389/fimmu.2026.1927894 (PMC13640461; doi:10.3389/fimmu.2026.1927894)
Supplement: Supplementary file 1 [file Supplementaryfile1.docx]

| **Characteristic** | **Overall (N=550)** | **Chongqing (N=180)** | **SWMU (N=370)** | **P-value** |
| --- | --- | --- | --- | --- |
| Age (years) | 63.00 (47.00, 72.00) | 64.50 (49.75, 73.00) | 62.50 (47.00, 71.00) | 0.21 |
| Height (cm) | 166.60 (160.70, 173.60) | 166.20 (160.90, 173.93) | 166.95 (160.60, 173.60) | 0.88 |
| Weight (kg) | 67.00 (60.62, 74.70) | 68.20 (61.03, 73.45) | 66.70 (60.60, 75.28) | 0.62 |
| Blood eosinophil count (×10⁹/L) | 0.12 (0.05, 0.31) | 0.12 (0.05, 0.31) | 0.11 (0.05, 0.31) | 0.77 |
| Eosinophil percentage (%) | 1.70 (0.90, 3.08) | 1.70 (0.90, 2.90) | 1.70 (0.90, 3.20) | 0.78 |
| FeNO (ppb) | 38.00 (17.00, 67.00) | 42.00 (18.50, 70.25) | 36.00 (17.00, 66.00) | 0.41 |
| ICS dose (µg/day) ᵃ | 160.00 (0.00, 320.00) | 160.00 (0.00, 320.00) | 160.00 (0.00, 320.00) | 0.44 |
| ACQ-5 score (0-6) ᵃ | 2.00 (1.00, 3.00) | 2.00 (1.00, 3.00) | 2.00 (1.00, 3.00) | 0.10 |
| FEV₁ %pred (%) | 87.90 (84.62, 91.80) | 87.05 (83.80, 91.23) | 88.25 (85.00, 91.97) | 0.07 |
| PEF variability (%) | 15.05 (9.30, 22.17) | 15.30 (8.62, 21.33) | 14.90 (9.55, 22.67) | 0.39 |
| FVC %pred (%) | 93.20 (87.60, 97.90) | 92.85 (86.20, 97.90) | 93.40 (88.32, 97.90) | 0.38 |
| Total IgE (IU/mL) | 32.50 (17.35, 62.05) | 33.00 (17.88, 62.98) | 32.50 (17.00, 61.25) | 0.57 |
| Specific IgE level (0-6) ᵃ | 0.00 (0.00, 0.00) | 0.00 (0.00, 0.00) | 0.00 (0.00, 0.00) | 0.49 |
| ICS response time (days) | 0.00 (0.00, 10.40) | 0.00 (0.00, 10.45) | 0.00 (0.00, 10.40) | 0.28 |
| ACT score | 23.00 (21.00, 25.00) | 23.00 (22.00, 25.00) | 24.00 (21.00, 25.00) | 0.43 |
| EQ-5D index | 0.98 (0.91, 1.00) | 0.97 (0.91, 1.00) | 0.99 (0.91, 1.00) | 0.60 |
| Exacerbation Status |  |  |  | 0.61 |
| No | 320 (58.2%) | 108 (60.0%) | 212 (57.3%) |  |
| Yes | 230 (41.8%) | 72 (40.0%) | 158 (42.7%) |  |
| Gender |  |  |  | 0.29 |
| Female | 270 (49.1%) | 82 (45.6%) | 188 (50.8%) |  |
| Male | 280 (50.9%) | 98 (54.4%) | 182 (49.2%) |  |
| Residence |  |  |  | 0.23 |
| Rural | 137 (24.9%) | 51 (28.3%) | 86 (23.2%) |  |
| Urban | 413 (75.1%) | 129 (71.7%) | 284 (76.8%) |  |
| Wheezing |  |  |  | 0.20 |
| No | 486 (88.4%) | 154 (85.6%) | 332 (89.7%) |  |
| Yes | 64 (11.6%) | 26 (14.4%) | 38 (10.3%) |  |
| Allergic rhinitis |  |  |  | 1.00 |
| No | 271 (49.3%) | 89 (49.4%) | 182 (49.2%) |  |
| Yes | 279 (50.7%) | 91 (50.6%) | 188 (50.8%) |  |
| Bronchodilator test positive |  |  |  | 1.00 |

**Table S1**. Baseline Characteristics by Center
