# Supplementary material for: Blood eosinophils as predictors of asthma exacerbations in adults: converging evidence from mendelian randomization, single-cell transcriptomics, and a two-center longitudinal cohort
Source: Front Immunol. 2026 Sep 22;17:1927894. doi: 10.3389/fimmu.2026.1927894 (PMC13640461; doi:10.3389/fimmu.2026.1927894)
Supplement: Supplementary file 2 [file Supplementaryfile2.docx]

Table S2. Stratified by center: baseline by outcome (Asthma Exacerbation).

|  | Chongqing | | | |  | SWMU | | | |
| --- | --- | --- | --- | --- | --- | --- | --- | --- | --- |
| Characteristic | Overall (N = 150) | No (N = 89) | Yes (N = 61) | p-value |  | Overall (N = 350) | No (N = 202) | Yes (N = 148) | p-value |
| Age (years) | 64.50 (49.75, 73.00) | 66.00 (50.75, 73.00) | 60.00 (45.50, 73.00) | 0.53 |  | 62.50 (47.00, 71.00) | 64.00 (47.75, 71.00) | 59.50 (46.00, 71.00) | 0.46 |
| Height (cm) | 166.20 (160.90, 173.93) | 166.65 (160.65, 175.15) | 165.45 (161.20, 171.72) | 0.45 |  | 166.95 (160.60, 173.60) | 167.10 (160.60, 173.12) | 166.75 (160.55, 174.28) | 0.78 |
| Weight (kg) | 68.20 (61.03, 73.45) | 67.80 (60.10, 73.30) | 68.80 (63.30, 73.88) | 0.61 |  | 66.70 (60.60, 75.28) | 67.00 (61.03, 75.90) | 65.55 (59.92, 73.58) | 0.18 |
| Blood eosinophil count (×10⁹/L) | 0.12 (0.05, 0.31) | 0.10 (0.04, 0.17) | 0.28 (0.06, 0.49) | <0.001 |  | 0.11 (0.05, 0.31) | 0.07 (0.04, 0.13) | 0.29 (0.11, 0.38) | <0.001 |
| Eosinophil percentage (%) | 1.70 (0.90, 2.90) | 1.50 (0.80, 2.10) | 2.65 (1.20, 3.20) | 0.002 |  | 1.70 (0.90, 3.20) | 1.30 (0.70, 1.90) | 3.05 (1.35, 3.60) | <0.001 |
| FeNO (ppb) | 42.00 (18.50, 70.25) | 43.00 (18.50, 77.25) | 38.50 (18.50, 63.50) | 0.64 |  | 36.00 (17.00, 66.00) | 35.00 (16.00, 64.25) | 38.00 (19.00, 76.75) | 0.11 |
| ICS dose (µg/day) ᵃ | 160.00 (0.00, 320.00) | 160.00 (0.00, 320.00) | 160.00 (160.00, 320.00) | 0.13 |  | 160.00 (0.00, 320.00) | 160.00 (0.00, 320.00) | 160.00 (0.00, 320.00) | 0.09 |
| ACQ-5 score (0-6) ᵃ | 2.00 (1.00, 3.00) | 2.00 (1.00, 3.00) | 2.00 (2.00, 3.00) | 0.026 |  | 2.00 (1.00, 3.00) | 2.00 (1.00, 2.00) | 2.00 (1.00, 3.00) | <0.001 |
| FEV₁ %pred (%) | 87.05 (83.80, 91.23) | 87.90 (85.18, 93.40) | 85.90 (81.27, 89.40) | 0.002 |  | 88.25 (85.00, 91.97) | 89.20 (85.88, 92.30) | 87.05 (84.12, 90.90) | <0.001 |
| PEF variability (%) | 15.30 (8.62, 21.33) | 14.80 (8.60, 21.33) | 16.30 (10.05, 21.43) | 0.41 |  | 14.90 (9.55, 22.67) | 14.10 (9.12, 22.02) | 16.25 (10.15, 23.80) | 0.14 |
| FVC %pred (%) | 92.85 (86.20, 97.90) | 94.30 (87.00, 99.12) | 90.25 (84.95, 96.15) | 0.014 |  | 93.40 (88.32, 97.90) | 94.05 (88.88, 99.03) | 92.40 (87.45, 96.78) | 0.024 |
| Total IgE (IU/mL) | 33.00 (17.88, 62.98) | 31.65 (18.10, 58.15) | 39.55 (17.82, 73.00) | 0.35 |  | 32.50 (17.00, 61.25) | 35.05 (18.45, 64.53) | 30.15 (16.32, 57.40) | 0.28 |
| Specific IgE level (0-6) ᵃ | 0.00 (0.00, 0.00) | 0.00 (0.00, 0.00) | 0.00 (0.00, 0.00) | 0.98 |  | 0.00 (0.00, 0.00) | 0.00 (0.00, 0.00) | 0.00 (0.00, 0.00) | 0.07 |
| ICS response time (days) | 0.00 (0.00, 10.45) | 0.00 (0.00, 8.62) | 0.00 (0.00, 11.40) | 0.048 |  | 0.00 (0.00, 10.40) | 0.00 (0.00, 10.20) | 0.00 (0.00, 10.67) | 0.14 |
| ACT score | 23.00 (22.00, 25.00) | 23.00 (22.00, 25.00) | 23.00 (21.00, 25.00) | 0.67 |  | 24.00 (21.00, 25.00) | 24.00 (22.00, 25.00) | 23.00 (21.00, 25.00) | 0.001 |
| EQ-5D index | 0.97 (0.91, 1.00) | 0.98 (0.93, 1.00) | 0.97 (0.88, 1.00) | 0.21 |  | 0.99 (0.91, 1.00) | 1.00 (0.93, 1.00) | 0.97 (0.88, 1.00) | 0.003 |
| Gender |  |  |  | 0.93 |  |  |  |  | 0.80 |
| Female | 82 (45.6%) | 50 (46.3%) | 32 (44.4%) |  |  | 188 (50.8%) | 106 (50.0%) | 82 (51.9%) |  |
| Male | 98 (54.4%) | 58 (53.7%) | 40 (55.6%) |  |  | 182 (49.2%) | 106 (50.0%) | 76 (48.1%) |  |
| Residence |  |  |  | 0.48 |  |  |  |  | 0.42 |
| Rural | 51 (28.3%) | 28 (25.9%) | 23 (31.9%) |  |  | 86 (23.2%) | 53 (25.0%) | 33 (20.9%) |  |
| Urban | 129 (71.7%) | 80 (74.1%) | 49 (68.1%) |  |  | 284 (76.8%) | 159 (75.0%) | 125 (79.1%) |  |
| Wheezing |  |  |  | 0.027 |  |  |  |  | 0.14 |
| No | 154 (85.6%) | 98 (90.7%) | 56 (77.8%) |  |  | 332 (89.7%) | 195 (92.0%) | 137 (86.7%) |  |
| Yes | 26 (14.4%) | 10 (9.3%) | 16 (22.2%) |  |  | 38 (10.3%) | 17 (8.0%) | 21 (13.3%) |  |
| Allergic rhinitis |  |  |  | 0.78 |  |  |  |  | 1.00 |
| No | 89 (49.4%) | 52 (48.1%) | 37 (51.4%) |  |  | 182 (49.2%) | 104 (49.1%) | 78 (49.4%) |  |
| Yes | 91 (50.6%) | 56 (51.9%) | 35 (48.6%) |  |  | 188 (50.8%) | 108 (50.9%) | 80 (50.6%) |  |
| Bronchodilator test positive |  |  |  | 1.00 |  |  |  |  | 0.18 |
| No | 180 (100.0%) | 108 (100.0%) | 72 (100.0%) |  |  | 368 (99.5%) | 212 (100.0%) | 156 (98.7%) |  |
| Yes | 0 (0.0%) | 0 (0.0%) | 0 (0.0%) |  |  | 2 (0.5%) | 0 (0.0%) | 2 (1.3%) |  |
