# Supplementary material for: Blood eosinophils as predictors of asthma exacerbations in adults: converging evidence from mendelian randomization, single-cell transcriptomics, and a two-center longitudinal cohort
Source: Front Immunol. 2026 Sep 22;17:1927894. doi: 10.3389/fimmu.2026.1927894 (PMC13640461; doi:10.3389/fimmu.2026.1927894)
Supplement: Supplementary file 3 [file Supplementaryfile3.docx]

**Table S3**. Sensitivity Analysis for ACQ-5, ICS dose and Specific IgE.

| **Characteristic** | **Overall (N=550)** | **No (N=320)** | **Yes (N=230)** | **P-value** |
| --- | --- | --- | --- | --- |
| ACQ-5 category (ATS/ERS) |  |  |  | <0.001 |
| Well-controlled (<0.75) | 92 (16.7%) | 69 (21.6%) | 23 (10.0%) |  |
| Partially controlled (0.75-1.5) | 93 (16.9%) | 58 (18.1%) | 35 (15.2%) |  |
| Poorly controlled (>1.5) | 365 (66.4%) | 193 (60.3%) | 172 (74.8%) |  |
| ICS dose (GINA 2023) |  |  |  | 0.07 |
| None (0) | 151 (27.5%) | 96 (30.0%) | 55 (23.9%) |  |
| Low (>0-400) | 348 (63.3%) | 201 (62.8%) | 147 (63.9%) |  |
| Medium (>400-800) | 51 (9.3%) | 23 (7.2%) | 28 (12.2%) |  |
| High (>800) | 0 (0.0%) | 0 (0.0%) | 0 (0.0%) |  |
| Specific IgE (CAP) |  |  |  | 0.20 |
| Negative (Class 0) | 490 (89.1%) | 280 (87.5%) | 210 (91.3%) |  |
| Positive (Class ≥1) | 60 (10.9%) | 40 (12.5%) | 20 (8.7%) |  |

Note: This sensitivity analysis presents categorical versions using clinically accepted thresholds. These categorical versions were NOT used in the machine-learning model. ACQ-5 thresholds: Reddel HK et al., Am J Respir Crit Care Med 2009. ICS dose thresholds: GINA 2023 adult budesonide-equivalent. Specific IgE: Hamilton RG, J Allergy Clin Immunol 2010.
