# Supplementary material for: Blood eosinophils as predictors of asthma exacerbations in adults: converging evidence from mendelian randomization, single-cell transcriptomics, and a two-center longitudinal cohort
Source: Front Immunol. 2026 Sep 22;17:1927894. doi: 10.3389/fimmu.2026.1927894 (PMC13640461; doi:10.3389/fimmu.2026.1927894)
Supplement: Supplementary file 4 [file Supplementaryfile4.docx]

**Table S4**. Discrimination and operating characteristics of the five machine-learning models and two benchmark comparators at Youden-optimal probability thresholds.

| Model | AUC (95% CI) | Threshold | Sensitivity (95% CI) | Specificity (95% CI) | PPV (95% CI) | NPV (95% CI) | Accuracy |
| --- | --- | --- | --- | --- | --- | --- | --- |
| Internal validation — SWMU (10-fold out-of-fold predictions) ᵃ | | | | | | | |
| Five-feature machine-learning models | | | | | | | |
| XGBoost | 0.876 (0.838–0.911) | 0.521 | 0.728 (0.665–0.797) | 0.901 (0.858–0.939) | 0.846 (0.793–0.901) | 0.816 (0.781–0.857) | 0.827 |
| RF | 0.875 (0.838–0.910) | 0.446 | 0.785 (0.722–0.842) | 0.821 (0.769–0.868) | 0.765 (0.713–0.818) | 0.837 (0.797–0.877) | 0.805 |
| SVM -RBF | 0.833 (0.789–0.871) | 0.528 | 0.741 (0.677–0.810) | 0.797 (0.745–0.849) | 0.731 (0.677–0.789) | 0.805 (0.767–0.848) | 0.773 |
| KNN | 0.823 (0.780–0.864) | 0.405 | 0.709 (0.639–0.778) | 0.821 (0.769–0.873) | 0.747 (0.692–0.806) | 0.791 (0.753–0.834) | 0.773 |
| LR | 0.801 (0.755–0.846) | 0.523 | 0.684 (0.614–0.753) | 0.863 (0.816–0.906) | 0.788 (0.732–0.848) | 0.785 (0.748–0.825) | 0.786 |
| Benchmark comparators | | | | | | | |
| 3-variable clinical model ᵇ | 0.765 (0.713–0.815) | 0.408 | 0.696 (0.627–0.766) | 0.807 (0.755–0.858) | 0.728 (0.673–0.790) | 0.781 (0.742–0.824) | 0.759 |
| Eosinophil count only ᶜ | 0.741 (0.686–0.797) | 0.445 | 0.665 (0.595–0.734) | 0.896 (0.854–0.934) | 0.827 (0.771–0.883) | 0.782 (0.748–0.821) | 0.797 |
| External validation — Chongqing cohort ᵈ | | | | | | | |
| Five-feature machine-learning models | | | | | | | |
| XGBoost | 0.769 (0.693–0.841) | 0.480 | 0.597 (0.486–0.708) | 0.843 (0.769–0.907) | 0.717 (0.621–0.815) | 0.758 (0.705–0.811) | 0.744 |
| RF | 0.763 (0.684–0.837) | 0.531 | 0.597 (0.486–0.708) | 0.852 (0.778–0.917) | 0.729 (0.632–0.827) | 0.760 (0.708–0.814) | 0.750 |
| LR | 0.755 (0.679–0.831) | 0.452 | 0.694 (0.583–0.792) | 0.769 (0.685–0.843) | 0.667 (0.583–0.754) | 0.790 (0.733–0.851) | 0.739 |
| SVM -RBF | 0.733 (0.652–0.809) | 0.518 | 0.694 (0.583–0.792) | 0.731 (0.639–0.815) | 0.633 (0.554–0.714) | 0.782 (0.722–0.845) | 0.717 |
| KNN | 0.718 (0.635–0.798) | 0.690 | 0.417 (0.306–0.528) | 0.963 (0.926–0.991) | 0.882 (0.774–0.972) | 0.712 (0.675–0.754) | 0.744 |
| Benchmark comparators | | | | | | | |
| 3-variable clinical model ᵇ | 0.733 (0.657–0.810) | 0.503 | 0.556 (0.444–0.667) | 0.852 (0.787–0.917) | 0.714 (0.618–0.814) | 0.742 (0.695–0.793) | 0.733 |
| Eosinophil count only ᶜ | 0.680 (0.596–0.765) | 0.528 | 0.528 (0.417–0.639) | 0.870 (0.806–0.926) | 0.731 (0.627–0.837) | 0.734 (0.688–0.784) | 0.733 |

AUC, area under the receiver-operating-characteristic curve; CI, confidence interval; LR, logistic regression; NPV, negative predictive value; PPV, positive predictive value; RBF, radial basis function; SVM, support vector machine; RF, Random Forest; KNN, k-nearest neighbours; LR, Logistic regression; SWMU, Southwest Medical University.

ᵃ Internal performance was estimated from 10-fold cross-validation out-of-fold predictions on the SWMU derivation cohort (n = 370).

ᵇ Multivariable logistic regression using age, FEV₁ % predicted, and blood eosinophil count.

ᶜ Univariable logistic regression using blood eosinophil count as the sole predictor.

ᵈ External performance was assessed by applying models refit on the full SWMU cohort to the independent Chongqing validation cohort (n = 180).

All 95% confidence intervals were computed via 2000 bootstrap resamples. Operating characteristics (sensitivity, specificity, PPV, NPV, accuracy) are reported at the Youden-index-optimal probability threshold determined on the corresponding validation set.
